# Use of an electronic patient-reported outcome measure in the management of patients with advanced chronic kidney disease: the RePROM pilot trial protocol

**DOI:** 10.1136/bmjopen-2018-026080

**Published:** 2018-10-28

**Authors:** Derek Kyte, Jon Bishop, Elizabeth Brettell, Melanie Calvert, Paul Cockwell, Mary Dutton, Helen Eddington, Gabby Hadley, Natalie J Ives, Louise J Jackson, Stephanie Stringer, Marie Valente

**Affiliations:** 1 Centre for Patient-Reported Outcomes Research, Institute of Applied Health Research, University of Birmingham, Birmingham, UK; 2 Birmingham Clinical Trials Unit (BCTU), Institute of Applied Health Research, University of Birmingham, Birmingham, UK; 3 Department of Renal Medicine, University Hospitals Birmingham NHS Foundation Trust, Birmingham, UK; 4 Health Economics Unit, Institute of Applied Health Research, University of Birmingham, Birmingham, UK

**Keywords:** end stage renal failure, chronic renal failure, patient-reported outcomes, symptom monitoring

## Abstract

**Introduction:**

Chronic kidney disease (CKD) affects up to 16% of adults in the UK. Patient quality of life is particularly reduced in end-stage renal disease and is strongly associated with increased hospitalisation and mortality. Thus, accurate and responsive healthcare is a key priority. Electronic patient-reported outcome measures (ePROMs) are online questionnaires which ask patients to self-rate their health status. Evidence in oncology suggests that the use of ePROM data within routine care, alongside clinical information, may enhance symptom management and improve patient outcomes. However, National Health Service (NHS)-based ePROM research in CKD is lacking. This pilot trial will assess the feasibility of undertaking a full-scale randomised controlled trial (RCT) in patients with CKD within the NHS.

**Methods and analysis:**

The renal ePROM pilot trial is an investigator-led single-centre, open-label, two-arm randomised controlled pilot trial of 66 participants ≥18 years with advanced CKD. Participants will be randomised to receive either usual care or usual care supplemented with an ePROM intervention. Participants within the intervention arm will be asked to submit monthly self-reports of their health status using the ePROM system. The system will provide tailored information to patients in response to each report and notify the clinical team of patient deterioration. The renal clinical team will monitor for ePROM notifications and will respond with appropriate action, in line with standard clinical practice. Measures of study feasibility, participant quality of life and CKD severity will be completed at 3 monthly intervals. Health economic outcomes will be assessed. Clinicians will record treatment decision-making. Acceptability and feasibility of the protocol will be assessed alongside outcome measure and intervention compliance rates. Qualitative process evaluation will be conducted.

**Ethics and dissemination:**

The findings will inform the design of a full-scale RCT and the results will be submitted for publication in peer-reviewed journals. The study has ethical approval.

**Trial registration numbers:**

ISRCTN12669006; Pre-results.

**Strengths and limitations of this study**

- The study uses a randomised controlled trial (RCT) design, delivered in a National Health Service (NHS) setting.
- The research site, University Hospitals Birmingham NHS Foundation Trust, hosts one of the largest specialist renal research sites in Europe.
- The study protocol and intervention was developed by a multidisciplinary team of experts including: patients, healthcare professionals and academics.
- As a pilot trial, the study is limited to one clinical centre and is not statistically powered to assess clinical outcomes. Instead, the project will evaluate feasibility of conducting a full-scale RCT in the target population.

## Introduction

Chronic kidney disease (CKD) affects up to 16% of adults in the UK,^1^ with an annual estimated cost of £1.45 billion.^2^ More than half of these costs were associated with renal replacement therapy (RRT)—haemodialysis, peritoneal dialysis and/or kidney transplantation—for patients with end-stage renal disease (ESRD).^2^ This is despite the fact that patients requiring RRT comprise less than 1% of the total CKD population.^2^ Numerous studies have demonstrated that patients with ESRD experience high symptom burden and a very high prevalence of depression.^3^ Moreover, patient quality of life is significantly reduced in ESRD and is strongly associated with increased hospitalisation and mortality.^3^ Accurate and responsive healthcare for patients as they progress from advanced CKD to ESRD is therefore a key healthcare priority.

Effective management of advanced CKD relies on the timely detection of clinical deterioration towards ESRD. This can be a major challenge between scheduled clinic visits, when it is often difficult to identify clinical deterioration unless a patient self-refers. Unfortunately, some patients self-refer too late because they have difficulty identifying the point at which they may require assistance. Without prompt recognition of advanced symptoms, such patients are at high risk of severe illness, emergency hospitalisation and associated worse clinical outcomes.^3^

Patient-reported outcome measures (PROMs) are validated questionnaires which ask patients to self-rate their health status. They can provide important information regarding the patient’s perspective on the physical, functional and psychological consequences of treatment and the degree and impact of disease symptoms.^4^ Evidence suggests that the use of PROM data, alongside regular clinical information, within routine care may:

- Aid patient–provider communication and support shared decision-making.^5^
- Improve patient activation and help patients to feel more involved/empowered in decisions around their care.^6–8^
- Improve the accuracy of symptom assessment and enhance symptom management.^9^
- Enhance patient education and self-management and maximise patient safety.^7 10–15^

With recent advances in technology, there has been considerable interest in the use of electronic PROMs (ePROMs) for the routine monitoring of patients with long-term conditions. ePROMs offer patients an ‘electronic’ method of data entry—for example, web based via PC, smartphone or using tablet devices—and give clinicians a flexible platform with which they may view PROM data.^14 16 17^

ePROMs offer patients the option of inputting data at a time and place, and via a platform, that is convenient to them. ePROM data can be used to help provide patients with tailored advice on self-management and can provide clinicians with detailed health-related quality of life (HRQL) and symptom data both in clinic and between scheduled appointments via home/remote data capture.^15^

For patients with advanced CKD, this would allow clinicians to monitor for symptom deterioration, facilitating the early detection of problems requiring attention and promoting timely intervention from the clinical team (eg, advice aimed at aiding patient self-management or escalation of care). Such intervention may delay disease progression and the need for costly and invasive RRT, and reduce emergency hospitalisations and other adverse outcomes.

A recent randomised controlled trial (RCT) conducted in an oncology setting in the USA, demonstrated that ePROM use is associated with improved HRQL, reduced accident and emergency visits, reduced hospitalisations and superior quality-adjusted survival.^15^ Both in Canada and Denmark, ePROM collection has been shown to be feasible in a renal population.^16 18 19^ However, National Health Service (NHS)-based ePROM research in CKD, utilising real-time patient and clinician feedback is lacking. Routine remote use of ePROM data by patients with advanced CKD may aid self-management, while also helping to improve the flow of information between patients and their clinicians, potentially improving patient safety, enhancing clinical interactions, optimising patient outcomes and delivering cost savings to the NHS.

A RCT is needed to evaluate ePROM efficacy in advanced CKD to determine if health professionals, providers and policy-makers should implement routine ePROM collection in renal practice. However, before a definitive trial is undertaken, a pilot trial is required to assess the feasibility of undertaking such a study and to help inform the key elements (eg, appropriate outcome measure, sample size) of the design for the full-scale RCT.

## Methods

### Design

The renal ePROM (RePROM) study will span two phases: stage 1 involves development of the ePROM intervention with patient and clinician input; stage 2 is a pilot/feasibility RCT with a qualitative substudy.

### Setting

Patients under the care of the renal services at Queen Elizabeth Hospital Birmingham (QEHB) will be recruited for this study.

#### Stage 1: intervention development

Design of the ePROM system will be finalised during a series of operational meetings, held in stage 1 of the study, with regular input from: (1) the QEHB renal clinical and research team; (2) the RePROM patient advisory group (PAG); (3) the QEHB IT and Informatics group; (4) the University of Birmingham Clinical Trials Unit (BCTU) and (5) the Patient CentredOutcomes Group at the University of Leeds.

The ePROM system will be made available to study participants via ‘myHealth@QEHB’, a secure patient portal linked to the patient’s electronic healthcare record (EHR), delivered by the host site. The system will be developed to allow patients to self-report their health status using a variety of electronic platforms, for example, PC, smartphone or tablet.

The ePROM system will be designed to: (1) provide appropriate self-management advice to participants whose questionnaire scores suggest mild/moderate symptoms and (2) notify the clinical team of patient deterioration via an automated email, where the patient’s questionnaire responses indicate severe symptoms/cause for concern.

Patients’ longitudinal ePROM scores will be made available to clinicians for use during routine outpatient consultations via the EHR. The RePROM PAG felt this approach would help to focus clinical discussion on patient-centred issues and may enhance symptom management, a view supported by related literature.^7 8 16 17 20–22^

#### Stage 2: pilot/feasibility RCT and qualitative substudy

##### Trial design

**Figure 1:**
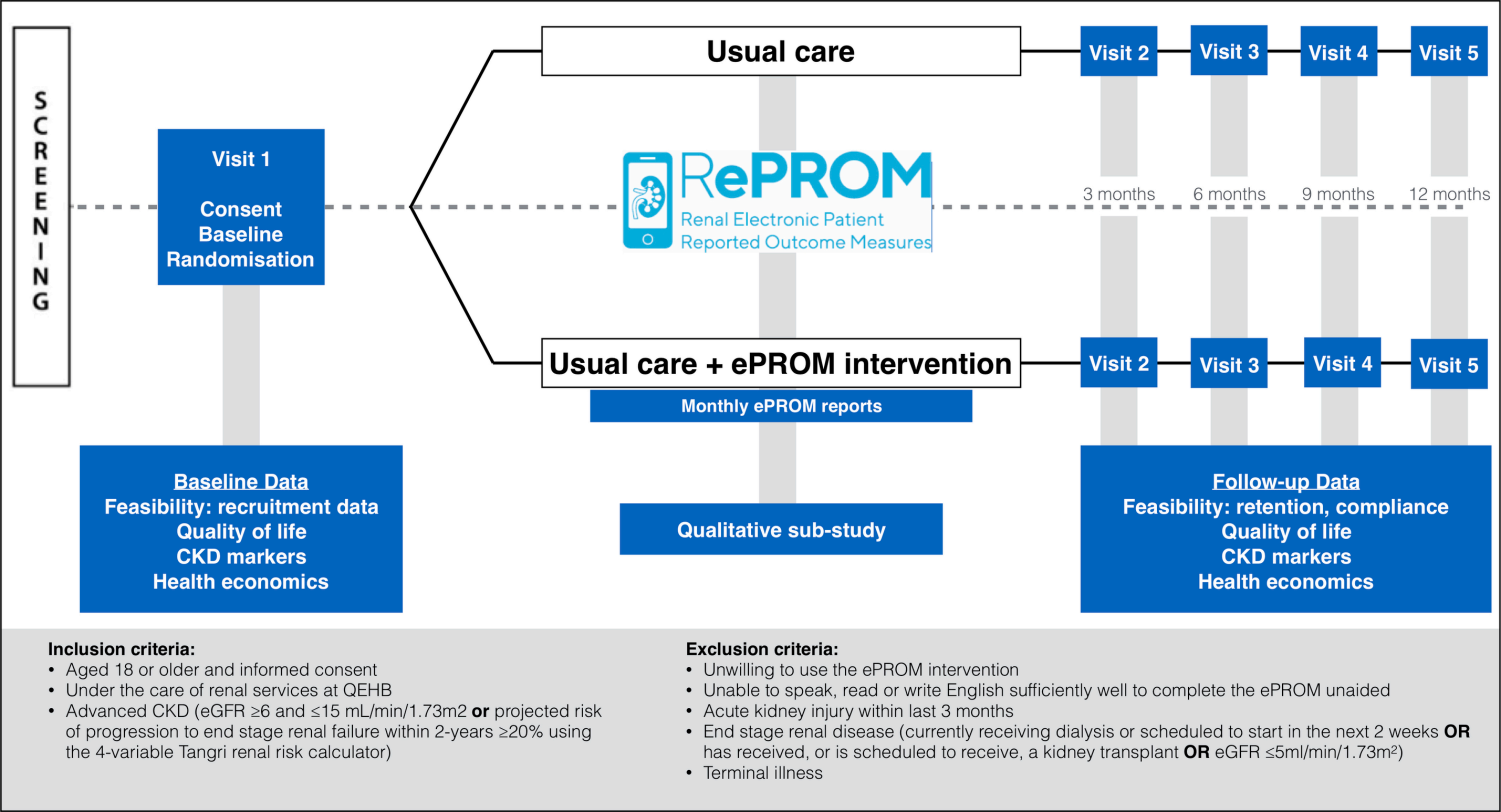
RePROM trial schema. CKD, chronic kidney disease; QEHB, Queen Elizabeth Hospital Birmingham.

The RePROM pilot trial is an investigator led single-centre, open-label, two-arm randomised controlled pilot trial of 66 participants aged 18 years or over with advanced CKD. Participants will be randomised to receive either usual care or usual care supplemented with an ePROM intervention. The study flow is outlined in figure 1.

### Recruitment and eligibility

Recruitment is scheduled to commence in July 2018. Patients will be invited to participate based on the following eligibility criteria:

#### Inclusion criteria

- Aged ≥18 years old.
- Ability to provide fully informed written consent for participation in the study.
- Patients under the care of the renal services at QEHB.
- Patients meeting the trial definition of advanced CKD:
  - An estimated Glomerular filtration rate (eGFR) ≥6 and ≤15 mL/min/1.73 m^2^ (inclusive).
  - **OR**
  - A projected risk of progression to end-stage renal failure within 2 years ≥20% using the 4-variable Tangri renal risk calculator.^23^

#### Exclusion criteria

- Patients unwilling to use the ePROM intervention.
- Patients who, in the opinion of the consenting professional, cannot speak, read or write English sufficiently well to complete the ePROM unaided.
- An episode of acute kidney injury (defined in accordance with national guidelines) within the last 3 months.^24^
- Patients meeting the trial definition of ESRD:
  - Currently receiving dialysis or scheduled to start in the next 2 weeks.
  - Has received (or has a scheduled date to receive) a kidney transplant.
  - eGFR ≤5 mL/min/1.73 m^2^.
- A terminal illness that, in the opinion of the consultant assessing eligibility, is likely to lead to the death of the patient within 6 months of starting participation in the study.

Patients fulfilling the entry criteria will have their eligibility assessed by qualified personnel at the host research site with access to/full understanding of their medical history. All patients approached will be given a copy of the participant information sheet (PIS). Usually, this will be sent to the patient in the post, along with an invitation letter, normally in advance of their next clinic assessment. The renal research team may also contact the patient by phone at the time of sending out the PIS. Staff will allow time for potential participants to consider the information provided, discuss the trial with their family and friends, and decide whether to take part. Alternatively, if deemed appropriate by the recruiting renal research team member, the PIS and invitation letter may be provided directly in clinic. Provided the patient feels they have had sufficient time to consider their potential involvement, consent may be sought at this same appointment.

Investigators/delegate(s) will ensure that they adequately explain the aim, trial intervention, anticipated benefits and potential hazards of taking part in the trial to the participant. They will also stress that participation is voluntary and that the participant is free to refuse to take part and may withdraw from the trial at any time. The participant will be given the opportunity to ask questions. If the participant expresses an interest in participating in the trial, they will be asked to sign and date the latest version of the informed consent form (ICF).

### Trial intervention

Participants in the control arm will continue to receive usual care. Participants randomised to the intervention arm will be asked to commence monthly self-reporting of their health status using the ePROM system, after receiving a face-to-face training session.

Participants will receive automated reminders prior to each scheduled self-report and 24 hours after a failure to report if necessary, these will be delivered via a secure myHealth QEHB patient portal email or text message, according to participants’ preferences. Participants may also upload additional ad hoc reports to the system, if they feel this is necessary (eg, if they wish to communicate a sudden change in symptoms).

The ePROM system will provide tailored information to patients in response to each report (both scheduled and ad hoc) and alert the clinical team of patient deterioration according to a priori determined alert threshold criteria established in the intervention development stage. After receiving training during the study setup period, the renal clinical team will monitor for ePROM alerts and will respond with appropriate clinical action, in line with standard clinical practice.

### Randomisation

Participants will be randomised at the level of the individual in a 1:1 ratio to either usual care (control arm) or usual care supplemented with an ePROM) system (experimental arm). A minimisation algorithm will be used within the online randomisation system to ensure balance in the treatment allocation over the following variables:

- Risk progression (<40%, vs ≥40%, using the 4-variable Tangri renal risk calculator^23^).
- Self-reported computer experience (defined as: regular use of a computer or tablet or smartphone at least weekly vs less than weekly).
- Ethnicity (‘white’ vs ‘non-white’).

A ‘random element’ will be included in the minimisation algorithm, so that each patient has a probability (unspecified here), of being randomised to the opposite treatment that they would have otherwise received.

### Outcome measures and study procedures

#### Primary outcome

The primary aims of the study are to pilot the trial protocol and assess the feasibility of undertaking a full-scale RCT on the use of ePROMs in the management of advanced CKD. This will include assessment of both quantitative and qualitative data. The pilot study will:

- Test and pilot the trial protocol (including recruitment and retention rates, data collection processes, data completeness and adherence to the ePROM intervention).
- Assess the willingness of clinicians to randomise participants into the trial.
- Assess the willingness of people with advanced CKD to be randomised into the trial.
- Assess the acceptability of the ePROM intervention;
- Explore the need for a non-web-based intervention platform for participants who are unable to use the ePROM.
- Inform selection of the most appropriate primary outcome measure for the full-scale RCT.
- Provide data to help estimate the sample size for the full-scale RCT.
- Provide a platform to develop and pilot the processes to capture costs and outcomes to inform the health economic evaluation for the full-scale RCT.
- Determine key participation criteria for centre involvement in the full-scale RCT.

#### Outcome data

This pilot trial is not powered to detect differences in outcome measures, but it provides the opportunity to ensure that there are no issues with completion of the outcome data and proposed outcome measures for the main RCT. The following outcome data will be collected:

- HRQL data, using the paper version of the EuroQol five dimension, five level, questionnaire (EQ-5D-5L). The EQ-5D-5L is a reliable/validated generic measure of health status/utility commonly used internationally in cost-effectiveness research.^25^
- Clinical data including: serum creatinine, calcium, phosphate, bicarbonate, albumin, eGFR, Albumin-to-creatinine ratio (ACR), blood pressure and for participants with diabetes: glucose and glycosylated haemoglobin (HbA1c).
- The following event data: progression to ESRD, contact with healthcare professionals in secondary care (outpatient clinics and accident and emergency), inpatient hospitalisation, death.
- Healthcare resource use data will be collected at each study visit.

All study staff/participants will be invited to complete a trial process questionnaire at the end of the study, which will evaluate aspects surrounding: data collection forms/questionnaires; randomisation procedure; acceptability of the intervention; appropriateness of the frequency of ePROM reporting; alert thresholds and management.

### Study procedures

During the baseline visit, patients will have demographic details recorded including age, self-assigned ethnicity, educational status, residential postcode (to derive Index of Multiple Deprivation), self-reported computer experience and medical history.

Patient quality of life will be assessed using a paper version of the EQ-5D-5L questionnaire (not a routine test). This will be completed by the participant at baseline and at 3, 6, 9 and 12 months postrandomisation (assessment window ±3 weeks). This questionnaire may be posted out to participants prior to their scheduled clinic/research visit, but research staff will be on hand in clinic to assist with completion where required. The EQ-5D-5L instrument is a reliable/validated generic measure of health status commonly used in CKD trials research.^25^

Clinical data will be collected at baseline and at 3, 6, 9 and 12 months (assessment window ±3 weeks), including: serum creatinine, calcium, phosphate, bicarbonate, albumin, eGFR, ACR, blood pressure and for participants with diabetes: glucose and HbA1c. Since these measures are routinely collected for clinical monitoring, the results closest to the calculated visit due date will be used for trial data, rather than repeating tests which have already been performed. If a result is not available within the visit window, the test should be performed at the trial visit. Healthcare resource use will be collected at 3, 6, 9 and 12 months (assessment window ±3 weeks).

All data will be extracted from the source records and entered onto a secure database maintained by the BCTU after each scheduled follow-up visit. The database will automatically capture ePROM notification data. Between clinic visits, patients will be managed in accordance with local practice.

### Sample size and justification

As this is a pilot trial, no formal sample size calculation has been performed. Following recommendations for pilot studies, 30 patients or more are typically required to obtain estimates of the parameters needed for sample size estimation.^26 27^ To allow for a 10% drop-out and loss to follow-up rate, this pilot trial will aim to recruit at least 33 participants in each group, a total of 66 participants. This will also allow the recruitment and retention rates to be estimated with 95% CI maximum widths of 20% and 25%, respectively.

### Analysis of outcome measures

A separate Statistical Analysis Plan will be produced and will provide a more comprehensive description of the planned statistical analyses. A brief outline of these analyses is given below.

The primary comparison groups will be composed of those randomised to usual care (control group) versus those randomised to usual care supplemented with the ePROM intervention (experimental group). In the first instance, all analyses will be based on the intention to treat principle, that is, all participants will be analysed in the treatment group to which they were randomised irrespective of compliance with the allocated treatment or other protocol deviation. The data analysis for this pilot trial will be descriptive and mainly focus on CI estimation, with no hypothesis testing performed.

Data will be explored to assess the key feasibility aspects of undertaking a full-scale RCT on the use of ePROMs in the management of advanced CKD. Dichotomous feasibility measures, such as the recruitment and retention rates, as well as data completeness, will be reported as numbers and percentages. Where appropriate, these values will be summarised across treatment groups.

Adherence with the ePROM intervention will be assessed by calculating the number and percentage of participants who complete the ePROM reports as scheduled. To be considered adherent, participants will need to have submitted their report within 72 hours of the scheduled time point. Incomplete submissions (ie, with some questions not answered) will be accepted for the purpose of measuring adherence. Ad hoc ePROM reports (ie, those completed outside the scheduled reporting periods) will not contribute to the assessment of adherence, although we will assess the number of ePROM reports completed by each participant.

The pilot data will also help inform the selection of the most appropriate primary outcome measure for the main RCT and provide data to facilitate estimation of the sample size required for the main RCT. Outcome data on HRQL and clinical data are collected at 3, 6, 9 and 12 months postrandomisation. Analysis methods will be chosen according to the data type of the outcome under investigation, in brief:

- Continuous endpoints (eg, quality of life): These data will be summarised using means and SDs, with differences in means with 95% CIs reported. Longitudinal plots of the data over time will also be constructed for visual presentation of the data.
- Categorical (dichotomous) endpoints (eg, hospitalisation rates): The number of participants and percentages experiencing the event will be summarised between groups.
- Time-to-event endpoints (eg, time to ESRD, mortality): The numbers of participants and percentages experiencing the event will be summarised over time between groups. Kaplan-Meier survival curves will be constructed for visual presentation of time-to-event data.

#### Subgroup analyses

Descriptive reports of subgroup variables will be limited to the same variables used in the minimisation algorithm. The availability and completeness of data for the subgroup variables will be summarised to assess their appropriateness as minimisation variables for the main trial, but no formal analysis will be undertaken.

#### Missing data and sensitivity analyses

Every attempt will be made to collect full follow-up data on all study participants; it is thus anticipated that missing data will be minimal. The assessment of missing data is an outcome measure of this pilot trial. If a suitable primary outcome is identified during the pilot trial, the level of missing data will form one component of the assessment of feasibility for a future trial. As this is a pilot trial, no formal sensitivity analysis will be conducted.

#### Planned interim analysis

As this is a pilot trial, there are no plans for undertaking any interim analyses. However, the trial steering committee (TSC) will have access to recruitment, retention and data collection information.

#### Planned final analyses

The primary analysis for the trial will occur once all participants have completed the 12-month assessment and corresponding outcome data has been entered onto the trial database, validated as being ready for analysis and the database locked. This analysis will include data items up to and including the 12-month assessment.

### Health economics

In this study, we will develop and pilot methods to capture the costs and outcomes to inform an economic evaluation in the main RCT. This will examine healthcare resource use and outcomes across the two arms of the study.

The primary perspective adopted will be NHS/personal social care; which will focus on healthcare resource use and costs including: renal staff activity in response to ePROM notifications; GP and hospital consultations; in-patient hospitalisation; medications; referrals and NHS costs associated with maintenance of the ePROM system.

Where possible, data on NHS resource utilisation will be collected from the electronic patient records. Other data will be collected via case report forms (CRFs), either completed in study follow-up visits or on event-triggered forms (eg, generated in response to an ePROM alert). Resource use will be valued using appropriate unit costs such as the British National Formulary and the most recent version of Unit Costs of Health and Social Care and NHS Reference Costs.^28^

### Monitoring

#### Management and oversight

The Trial Management Group will monitor all aspects of the conduct and progress of the trial, ensure that the protocol is adhered to and take appropriate action to safeguard participants and the quality of the trial itself.

A single TSC will be convened and will meet at least yearly and as required depending on the needs of the TSC/trial office. The TSC will provide overall oversight of the trial, including the practical aspects of the study, as well as ensuring that the study is run in a way which is both safe for the patients and provides appropriate feasibility data to the sponsor and investigators. The TSC will also undertake the final assessment of feasibility. Given this is a pilot study, a data monitoring committee is not required.

### Adverse events

The collection and reporting of adverse events (AEs) will be in accordance with the UK Policy Framework for Health and Social Care Research and the requirements of the Health Research Authority. The Investigator will assess the seriousness and causality (relatedness) of all AEs experienced by the trial participants and this assessment will be documented in the source data, with reference to the protocol.

### Audit

Trials staff will be in regular contact with the site research team to check on progress and address any queries that they may have. Trials staff will check incoming ICFs and CRFs for compliance with the protocol, data consistency, missing data and timing. Sites will be sent data clarification forms requesting missing data or clarification of inconsistencies or discrepancies. Sites will be requested to send in copies of signed ICFs and other documentation for in-house review for all participants providing explicit consent.

### Qualitative substudy

#### Objective

To explore patient and study personnel/clinician thoughts/experiences regarding the RePROM trial processes.

#### Methods

Participants and study personnel/clinicians involved in the pilot trial will be invited to take part in the qualitative substudy. Up to 40 participants (20 patients and 20 study personnel/clinicians) will be recruited, purposively selected to capture those participants who experienced a range of outcomes and experiences during the trial where possible. However, recruitment will continue until data saturation is reached.

Semistructured interviews will be conducted by a member of the Centre for Patient-Reported Outcome Research team at the University of Birmingham according to a predefined topic guide, but there will be sufficient scope to explore novel themes where appropriate. All interviews will be digitally recorded, professionally transcribed and the transcripts anonymised. Transcript data will be entered into a specialist software package (eg, NVivo, QSR International) to aid organisation and analysis of the data. All data will be analysed by the CI using conventional content analysis. Formal triangulation of coding and member checking will be employed to enhance the credibility of the analysis. Only anonymised quotes will be used in any arising publications or reports.

## Ethics, confidentiality and dissemination

Personal data recorded on all documents will be regarded as strictly confidential and will be handled and stored in accordance with the Data Protection Act 1998. Data transferred from the host site to the researchers (University of Birmingham) will be securely stored and only used for analysis or study monitoring relevant to the participant taking part in the research. The findings from the trial will be used to inform the design of a future, full-scale, RCT. Results will be submitted for publication in a peer-reviewed journal, presented at scientific conferences and disseminated to the public via lay summaries/newsletters in partnership with our PAG. Study manuscripts will be prepared by the chief investigator and authorship will be determined by the trial publication policy. Intellectual property rights will be addressed in the Clinical Study Site Agreement between Sponsor and site. The full protocol is available on request to the corresponding author.

## Patient and public involvement

Development of the research question, outcome measures and study design was informed by a series of meetings held with the RePROM PAG, which included people with lived experience of CKD. The PAG reviewed all patient-facing study documentation and considered the overall burden of study participation during the design process.

## Supplementary Material

Reviewer comments

Author's manuscript
