# Supplementary material for: Use of an electronic patient-reported outcome measure in the management of patients with advanced chronic kidney disease: the RePROM pilot trial protocol
Source: BMJ Open. 2018 Oct 28;8(10):e026080. doi: 10.1136/bmjopen-2018-026080 (PMC6224762; doi:10.1136/bmjopen-2018-026080)
Supplement: Author's manuscript [file bmjopen-2018-026080.draft_revisions.pdf]

BMJ Open

BMJ Open is committed to open peer review. As part of this commitment we make the peer review history of every article we publish publicly available.

When an article is published we post the peer reviewers' comments and the authors' responses online. We also post the versions of the paper that were used during peer review. These are the versions that the peer review comments apply to.

The versions of the paper that follow are the versions that were submitted during the peer review process. They are not the versions of record or the final published versions. They should not be cited or distributed as the published version of this manuscript.

BMJ Open is an open access journal and the full, final, typeset and author-corrected version of record of the manuscript is available on our site with no access controls, subscription charges or pay-per-view fees (http://bmjopen.bmj.com).

If you have any questions on BMJ Open's open peer review process please email
info.bmjopen@bmj.com

BMJ Open

The use of an electronic Patient-Reported Outcome Measure in the Management of Patients with Advanced Chronic Kidney Disease. The RePROM Pilot Trial.

Journal:	*BMJ Open*
Manuscript ID	bmjopen-2018-026080
Article Type:	Protocol
Date Submitted by the Author:	21-Aug-2018
Complete List of Authors:	Kyte, Derek; University of Birmingham, Centre for Patient-Reported Outcomes Research, Institute of Applied Health Research<br>Bishop, Jon; NIHR Surgical Reconstruction and Microbiology Research Centre, Medical Statistician<br>Brettell, Elizabeth; 1. Birmingham Clinical Trials Unit (BCTU), Institute of Applied Health Research University of Birmingham, Birmingham, UK<br>Calvert, Melanie; The University of Birmingham, Centre for Patient-Reported Outcomes Research, Institute of Applied Health Research<br>Cockwell, Paul; University Hospitals Birmingham NHS Foundation Trust, Renal Medicine<br>Dutton, Mary; University Hospitals Birmingham NHS Foundation Trust, Renal Medicine<br>Eddington, Helen; University Hospitals Birmingham NHS Foundation Trust, Renal Medicine<br>Hadley, Gabby; University Hospitals Birmingham NHS Foundation Trust, Renal Medicine<br>Ives, Natalie; 1. Birmingham Clinical Trials Unit (BCTU), Institute of Applied Health Research University of Birmingham, Birmingham, UK<br>Jackson, Louise; University of Birmingham, Health Economics Unit<br>Stringer, Stephanie; University Hospitals Birmingham NHS Foundation Trust, Renal Medicine<br>Valente, Marie; 1. Birmingham Clinical Trials Unit (BCTU), Institute of Applied Health Research University of Birmingham, Birmingham, UK
Keywords:	End stage renal failure < NEPHROLOGY, Chronic renal failure < NEPHROLOGY, patient-reported outcomes, symptom monitoring

SCHOLARONE™
Manuscripts

TITLE

The use of an electronic Patient-Reported Outcome Measure in the Management of Patients with Advanced Chronic Kidney Disease. The RePROM Pilot Trial.

AUTHORSHIP

Derek Kyte[1], Jon Bishop[2], Elizabeth Brettell[2], Melanie Calvert[1], Paul Cockwell[3], Mary Dutton[3], Helen Eddington[3], Gabby Hadley[3], Natalie Ives[2], Louise Jackson[4], Stephanie Stringer[3], Marie Valente[2].

AUTHOR AFFILIATIONS

1. Centre for Patient-Reported Outcomes Research, Institute of Applied Health Research, University of Birmingham, Birmingham, UK
2. Birmingham Clinical Trials Unit (BCTU), Institute of Applied Health Research University of Birmingham, Birmingham, UK
3. University Hospitals Birmingham NHS Foundation Trust, Birmingham, UK
4. Health Economics Unit, Institute of Applied Health Research, University of Birmingham, Birmingham, UK

CORRESPONDING AUTHOR

Dr Derek Kyte
Lecturer in Health Research Methods
The Murray Learning Centre
University of Birmingham
Birmingham
B15 2TT

Telephone: 0121 415 8502
Email: d.g.kyte@bham.ac.uk

WORD COUNT

3994

KEYWORDS

Chronic Kidney Diseases, Patient-reported outcomes, symptom monitoring

For peer review only - http://bmjopen.bmj.com/site/about/guidelines.xhtml

ABSTRACT

Introduction

Chronic Kidney Disease (CKD) affects up to 16% of adults in the UK. Patient quality of life is particularly reduced in end stage renal disease and is strongly associated with increased hospitalisation and mortality. Thus, accurate and responsive healthcare is a key priority. Electronic Patient-Reported Outcome Measures (ePROMs) are online questionnaires which ask patients to self-rate their health status. Evidence in oncology suggests the use of ePROM data within routine care, alongside clinical information, may enhance symptom management and improve patient outcomes. However, NHS-based ePROM research in CKD is lacking. This pilot trial will assess the feasibility of undertaking a full-scale randomised controlled trial in patients with CKD within the NHS.

Methods and analysis

The RePROM pilot trial is an investigator-led single-centre, open-label, two-arm randomised controlled pilot trial of 66 participants ≥18 years with advanced CKD. Participants will be randomised to receive either usual care, or usual care supplemented with an ePROM intervention. Participants within the intervention arm will be asked to submit monthly self-reports of their health status using the ePROM system. The system will provide tailored information to patients in response to each report and notify the clinical team of patient deterioration. The renal clinical team will monitor for ePROM notifications and will respond with appropriate action, in line with standard clinical practice. Measures of study feasibility, participant quality of life and CKD severity will be completed at 3-monthly intervals. Health economic outcomes will be assessed. Clinicians will record treatment decision-making. Acceptability and feasibility of the protocol will be assessed alongside outcome measure and intervention compliance rates. Qualitative process evaluation will be conducted.

Ethics and dissemination

The findings will inform the design of a full-scale RCT and the results will be submitted for publication in peer-reviewed journals. The study has ethical approval.

Trial registration number

ISRCTN12669006

For peer review only - http://bmjopen.bmj.com/site/about/guidelines.xhtml

ARTICLE SUMMARY

Strengths and limitations of this study

- The study uses a randomised controlled trial design, delivered in an NHS setting.
- The research site, University Hospitals Birmingham NHS Foundation Trust, hosts one of the largest specialist renal research sites in Europe.
- The study protocol and intervention was developed by a multidisciplinary team of experts including: patients, health care professionals and academics.
- As a pilot trial, the study is limited to one clinical centre and is not statistically powered to assess clinical outcomes. Instead, the project will evaluate feasibility of conducting a full-scale RCT in the target population.

For peer review only

For peer review only - http://bmjopen.bmj.com/site/about/guidelines.xhtml

INTRODUCTION

Chronic Kidney Disease (CKD) affects up to 16% of adults in the UK[1], with an annual estimated cost of £1.45 billion.[2] More than half of these costs were associated with Renal Replacement Therapy (RRT) - haemodialysis, peritoneal dialysis and/or kidney transplantation - for patients with end stage renal disease (ESRD).[2] This is despite the fact that patients requiring RRT comprise less than 1% of the total CKD population.[2] Numerous studies have demonstrated that patients with ESRD experience high symptom burden and a very high prevalence of depression.[3] Moreover, patient quality of life is significantly reduced in ESRD and is strongly associated with increased hospitalisation and mortality.[3] Accurate and responsive healthcare for patients as they progress from advanced CKD to ESRD is therefore a key healthcare priority.

Effective management of advanced CKD relies on the timely detection of clinical deterioration towards ESRD. This can be a major challenge between scheduled clinic visits, when it is often difficult to identify clinical deterioration unless a patient self-refers. Unfortunately, some patients self-refer too late because they have difficulty identifying the point at which they may require assistance. Without prompt recognition of advanced symptoms, such patients are at high risk of severe illness, emergency hospitalisation, and associated worse clinical outcomes.[3]

Patient-Reported Outcome Measures (PROMs) are validated questionnaires which ask patients to self-rate their health status. They can provide important information regarding the patient's perspective on the physical, functional and psychological consequences of treatment and the degree and impact of disease symptoms.[4] Evidence suggests the use of PROM data, alongside regular clinical information, within routine care may:

- Aid patient-provider communication and support shared decision-making.[5]
- Improve patient activation and help patients to feel more involved/empowered in decisions around their care.[6-8]
- Improve the accuracy of symptom assessment and enhance symptom management.[9]
- Enhance patient education and self-management and maximise patient safety.[7 10-15]

With recent advances in technology, there has been considerable interest in the use of electronic PROMs (ePROMs) for the routine monitoring of patients with long-term conditions. ePROMs offer patients an 'electronic' method of data entry – e.g. web-based via PC, smartphone, or using tablet devices – and give clinicians a flexible platform with which they may view PROM data.[14 16 17]

ePROMs offer patients the option of inputting data at a time and place, and via a platform, that is convenient to them. ePROM data can be used to help provide patients with tailored advice on self-management and can provide clinicians with detailed HRQL and symptom data both in-clinic and between scheduled appointments via home/remote data capture.[15]

For peer review only - http://bmjopen.bmj.com/site/about/guidelines.xhtml

For patients with advanced CKD, this would allow clinicians to monitor for symptom deterioration, facilitating the early detection of problems requiring attention and promoting timely intervention from the clinical team (e.g. advice aimed at aiding patient self-management or escalation of care). Such intervention may delay disease progression and the need for costly and invasive RRT, and reduce emergency hospitalisations and other adverse outcomes.

A recent randomised controlled trial (RCT) conducted in an oncology setting in the US, demonstrated that ePROM use is associated with improved HRQL, reduced Accident and Emergency visits, reduced hospitalisations, and superior quality-adjusted survival.[15] Both in Canada and Denmark, ePROM collection has been shown to be feasible in a renal population.[16 18 19] However, NHS-based ePROM research in CKD, utilising real-time patient and clinician feedback is lacking. Routine remote use of ePROM data by patients with advanced CKD may aid self-management, whilst also helping to improve the flow of information between patients and their clinicians, potentially improving patient safety, enhancing clinical interactions, optimising patient outcomes and delivering cost savings to the NHS.

A RCT is needed to evaluate ePROM efficacy in advanced CKD to determine if health professionals, providers and policy-makers should implement routine ePROM collection in renal practice. However, before a definitive trial is undertaken, a pilot trial is required to assess the feasibility of undertaking such a study and to help inform the key elements (e.g. appropriate outcome measure, sample size) of the design for the full-scale RCT.

For peer review only

For peer review only - http://bmjopen.bmj.com/site/about/guidelines.xhtml

METHODS

Design

The RePROM study will span two phases: stage 1 involves development of the ePROM intervention with patient and clinician input; stage 2 is a pilot/feasibility RCT with a qualitative sub-study.

Setting

Patients under the care of the renal services at Queen Elizabeth Hospital, Birmingham (QEHB) will be recruited for this study.

Stage 1: Intervention development

Design of the ePROM system will be finalised during a series of operational meetings, held in stage 1 of the study, with regular input from: (1) the QEHB renal clinical and research team; (2) the RePROM Patient Advisory Group; (3) the QEHB IT and Informatics group; (4) the University of Birmingham Clinical Trials Unit; and (5) the Patients Outcomes Group at the University of Leeds.

The ePROM system will be made available to study participants via 'myHealth@QEHB', a secure patient portal linked to the patient's electronic healthcare record (EHR), delivered by the host site. The system will be developed to allow patients to self-report their health status using a variety of electronic platforms, e.g.: PC, smartphone or tablet.

The ePROM system will be designed to: (i) provide appropriate self-management advice to participants whose questionnaire scores suggest mild/moderate symptoms; and (ii) notify the clinical team of patient deterioration via an automated email, where the patient's questionnaire responses indicate severe symptoms/cause for concern.

Patients' longitudinal ePROM scores will be made available to clinicians for use during routine outpatient consultations via the EHR. The RePROM Patient Advisory Group felt this approach would help to focus clinical discussion on patient-centred issues and may enhance symptom management, a view supported by related literature.[7 8 16 17 21-23]

Stage 2: pilot/feasibility RCT and qualitative sub-study

Trial design

The RePROM pilot trial is an investigator led single-centre, open-label, two-arm randomised controlled pilot trial of 66 participants aged 18 years or over with advanced CKD. Participants will be randomised to receive either usual care, or usual care supplemented with an ePROM intervention. The trial is registered with ISRCTN (ISRCTN12669006) and the NIHR Portfolio (CPMS ID: 36497). The study flow is outlined in Figure 1.

Figure 1. RePROM trial schema.

For peer review only - http://bmjopen.bmj.com/site/about/guidelines.xhtml

Recruitment and eligibility
Recruitment is scheduled to commence in July 2018. Patients will be invited to participate based on the following eligibility criteria:

Inclusion Criteria
- Aged ≥18 years old;
- Ability to provide fully informed written consent for participation in the study;
- Patients under the care of the renal services at QEHB;
- Patients meeting the trial definition of **advanced CKD**:
  - an eGFR ≥6 and ≤15 mL/min/1.73m^2 (inclusive)

  OR
  - a projected risk of progression to end-stage renal failure within 2-years ≥20% using the 4-variable Tangri renal risk calculator.[24]

Exclusion Criteria
- Patients unwilling to use the ePROM intervention;
- Patients who, in the opinion of the consenting professional, cannot speak, read or write English sufficiently well to complete the ePROM unaided;
- An episode of **acute kidney injury** (defined in accordance with national guidelines) within the last 3 months[25];
- Patients meeting the trial definition of **End Stage Renal Disease**:
  - Currently receiving dialysis or scheduled to start in the next 2 weeks

  OR
  - Has received (or has a scheduled date to receive) a kidney transplant

  OR
  - eGFR ≤5ml/min/1.73m^2;
- A terminal illness that, in the opinion of the consultant assessing eligibility, is likely to lead to the death of the patient within 6 months of starting participation in the study.

Patients fulfilling the entry criteria will have their eligibility assessed by qualified personnel at the host research site with access to/full understanding of their medical history. All patients approached will be given a copy of the participant information sheet (PIS). Usually, this will be sent to the patient in the post, along with an invitation letter, normally in advance of their next clinic assessment. The renal research team may also contact the patient by phone at the time of sending out the PIS. Staff will allow time for potential participants to consider the information provided, discuss the trial with their family and friends, and decide whether to take part. Alternatively, if deemed appropriate by the recruiting renal research team member, the PIS and invitation letter may be provided directly in clinic. Provided the patient feels they have had sufficient time to consider their potential involvement, consent may be sought at this same appointment.

For peer review only

For peer review only - http://bmjopen.bmj.com/site/about/guidelines.xhtml

Investigators/delegate(s) will ensure that they adequately explain the aim, trial intervention, anticipated benefits and potential hazards of taking part in the trial to the participant. They will also stress that participation is voluntary and that the participant is free to refuse to take part and may withdraw from the trial at any time. The participant will be given the opportunity to ask questions. If the participant expresses an interest in participating in the trial, they will be asked to sign and date the latest version of the Informed Consent Form.

Trial intervention

Participants in the control arm will continue to receive usual care. Participants randomised to the intervention arm will be asked to commence monthly self-reporting of their health status using the ePROM system, after receiving a face-to-face training session.

Participants will receive automated reminders prior to each scheduled self-report and 24 hours after a failure to report if necessary, these will be delivered via a secure myHealth QEHB patient portal email or text message, according to participants' preferences. Participants may also upload additional ad-hoc reports to the system, if they feel this is necessary (e.g.: if they wish to communicate a sudden change in symptoms).

The ePROM system will provide tailored information to patients in response to each report (both scheduled and ad-hoc) and alert the clinical team of patient deterioration according to *a priori* determined alert threshold criteria established in the intervention development stage. After receiving training during the study setup period, the renal clinical team will monitor for ePROM alerts and will respond with appropriate clinical action, in line with standard clinical practice.

Randomisation

Participants will be randomised at the level of the individual in a 1:1 ratio to either usual care (control arm) or usual care supplemented with an electronic Patient-Reported Outcome Measure (ePROM) system (experimental arm). A minimisation algorithm will be used within the online randomisation system to ensure balance in the treatment allocation over the following variables:

- Risk progression (<40%, versus ≥40%, using the 4-variable Tangri renal risk calculator[24]);
- Self-reported computer experience (defined as: regular use of a computer or tablet or smartphone at least weekly versus less than weekly);
- Ethnicity ('white' versus 'non-white')

A 'random element' will be included in the minimisation algorithm, so that each patient has a probability (unspecified here), of being randomised to the opposite treatment that they would have otherwise received.

Outcome measures and study procedures

Primary outcome

For peer review only - http://bmjopen.bmj.com/site/about/guidelines.xhtml

The primary aims of the study are to pilot the trial protocol and assess the feasibility of undertaking a full-scale RCT on the use of ePROMs in the management of advanced CKD. This will include assessment of both quantitative and qualitative data. The pilot study will:

- Test and pilot the trial protocol (including recruitment and retention rates, data collection processes, data completeness and adherence to the ePROM intervention);
- Assess the willingness of clinicians to randomise participants into the trial;
- Assess the willingness of people with advanced CKD to be randomised into the trial;
- Assess the acceptability of the ePROM intervention;
- Explore the need for a non-web-based intervention platform for participants who are unable to use the ePROM;
- Inform selection of the most appropriate primary outcome measure for the full-scale RCT;
- Provide data to help estimate the sample size for the full-scale RCT;
- Provide a platform to develop and pilot the processes to capture costs and outcomes to inform the health economic evaluation for the full-scale RCT;
- Determine key participation criteria for centre involvement in the full-scale RCT.

Outcome data

This pilot trial is not powered to detect differences in outcome measures, but it provides the opportunity to ensure that there are no issues with completion of the outcome data and proposed outcome measures for the main RCT. The following outcome data will be collected:

- HRQL data, using the paper version of the EQ-5D-5L. The EQ-5D-5L is a reliable/validated generic measure of health status/utility commonly used internationally in cost-effectiveness research.[26]
- Clinical data, including: Serum Creatinine, Calcium, Phosphate, Bicarbonate, Albumin, eGFR, ACR, blood pressure, and for participants with diabetes: glucose and HbA1c.
- The following event data: progression to end stage renal disease, contact with health care professionals in secondary care (outpatient clinics and Accident and Emergency); inpatient hospitalisation; death.
- Healthcare resource use data will be collected at each study visit.

All study staff/participants will be invited to complete a trial process questionnaire at the end of the study, which will evaluate aspects surrounding: data collection forms/questionnaires; randomisation procedure; acceptability of the intervention; appropriateness of the frequency of ePROM reporting; alert thresholds and management.

For peer review only - http://bmjopen.bmj.com/site/about/guidelines.xhtml

Study procedures

During the baseline visit, patients will have demographic details recorded including age, self-assigned ethnicity, educational status, residential postcode (to derive Index of Multiple Deprivation), self-reported computer experience and medical history.

Patient quality of life will be assessed using a paper version of the EQ-5D-5L questionnaire (not a routine test). This will be completed by the participant at baseline, and at 3, 6, 9 and 12 months post randomisation (assessment window +/- 3 weeks). This questionnaire may be posted out to participants prior to their scheduled clinic/research visit, but research staff will be on hand in clinic to assist with completion where required. The EQ-5D-5L instrument is a reliable/validated generic measure of health status commonly used in CKD trials research.[26]

Clinical data will be collected at baseline, and at 3, 6, 9 and 12 months (assessment window +/- 3 weeks), including: Serum Creatinine, Calcium, Phosphate, Bicarbonate, Albumin, eGFR, ACR, blood pressure, and for participants with diabetes: glucose and HbA1c. Since these measures are routinely collected for clinical monitoring, the results closest to the calculated visit due date will be used for trial data, rather than repeating tests which have already been performed. If a result is not available within the visit window, the test should be performed at the trial visit. Healthcare resource use will be collected at 3, 6, 9 and 12 months (assessment window +/- 3 weeks).

All data will be extracted from the source records and entered onto a secure database maintained by the Birmingham Clinical Trials Unit (BCTU) after each scheduled follow-up visit. The database will automatically capture ePROM notification data. Between clinic visits, patients will be managed in accordance with local practice.

Sample size and justification

As this is a pilot trial, no formal sample size calculation has been performed. Following recommendations for pilot studies, 30 patients or more are typically required to obtain estimates of the parameters needed for sample size estimatation.[27 28] To allow for a 10% drop-out and lost to follow-up rate, this pilot trial will aim to recruit at least 33 participants in each group, a total of 66 participants. This will also allow the recruitment and retention rates to be estimated with 95% confidence interval maximum widths of 20% and 25% respectively.

Analysis of Outcome Measures

A separate Statistical Analysis Plan will be produced and will provide a more comprehensive description of the planned statistical analyses. A brief outline of these analyses is given below.

The primary comparison groups will be composed of those randomised to usual care (control group) versus those randomised to usual care supplemented with the ePROM intervention (experimental group). In the first instance, all analyses will be based on the intention to treat principle, i.e. all participants will be analysed in the treatment group to which they were randomised irrespective of compliance with the allocated treatment or other protocol deviation. The data analysis for this pilot trial

For peer review only - http://bmjopen.bmj.com/site/about/guidelines.xhtml

will be descriptive and mainly focus on confidence interval estimation, with no hypothesis testing performed.

Data will be explored to assess the key feasibility aspects of undertaking a full-scale RCT on the use of ePROMs in the management of advanced CKD. Dichotomous feasibility measures, such as the recruitment and retention rates, as well as data completeness will be reported as numbers and percentages. Where appropriate, these values will be summarised across treatment groups.

Adherence with the ePROM intervention will be assessed by calculating the number and percentage of participants who complete the ePROM reports as scheduled. To be considered adherent, participants will need to have submitted their report within 72 hours of the scheduled time-point. Incomplete submissions (i.e. with some questions not answered) will be accepted for the purpose of measuring adherence. Ad-hoc ePROM reports (i.e. those completed outside the scheduled reporting periods) will not contribute to the assessment of adherence, although we will assess the number of ePROM reports completed by each participant.

The pilot data will also help inform the selection of the most appropriate primary outcome measure for the main RCT and provide data to facilitate estimation of the sample size required for the main RCT. Outcome data on HRQL and clinical data are collected at 3, 6, 9 and 12 months post-randomisation. Analysis methods will be chosen according to the data type of the outcome under investigation, in brief:

- *Continuous endpoints (e.g. quality of life)*: These data will be summarised using means and standard deviations, with differences in means with 95% confidence intervals reported. Longitudinal plots of the data over time will also be constructed for visual presentation of the data.
- *Categorical (dichotomous) endpoints (e.g. hospitalisation rates)*: The number of participants and percentages experiencing the event will be summarised between groups.
- *Time to Event endpoints (e.g. time to ESRD, mortality):* The numbers of participants and percentages experiencing the event will be summarised over time between groups. Kaplan-Meier survival curves will be constructed for visual presentation of time-to-event data.

Subgroup Analyses

Descriptive reports of subgroup variables will be limited to the same variables used in the minimisation algorithm. The availability and completeness of data for the subgroup variables will be summarised to assess their appropriateness as minimisation variables for the main trial, but no formal analysis will be undertaken.

Missing data and sensitivity analyses

Every attempt will be made to collect full follow-up data on all study participants; it is thus anticipated that missing data will be minimal. The assessment of missing data is an outcome measure of this pilot trial. If a suitable primary outcome is identified during the pilot trial, the level of missing data will form one component of the assessment of feasibility for a future trial. As this is a pilot trial, no formal sensitivity analysis will be conducted.

For peer review only - http://bmjopen.bmj.com/site/about/guidelines.xhtml

Planned interim analysis
As this is a pilot trial, there are no plans for undertaking any interim analyses. However, the Trial Steering Committee (TSC) will have access to recruitment, retention and data collection information.

Planned final analyses
The primary analysis for the trial will occur once all participants have completed the 12 month assessment and corresponding outcome data has been entered onto the trial database, validated as being ready for analysis, and the database locked. This analysis will include data items up to and including the 12 month assessment.

Health economics
In this study, we will develop and pilot methods to capture the costs and outcomes to inform an economic evaluation in the main RCT. This will examine healthcare resource use and outcomes across the two arms of the study.

The primary perspective adopted will be NHS/personal social care; which will focus on healthcare resource use and costs including: renal staff activity in response to ePROM notifications; GP and hospital consultations; in-patient hospitalisation; medications; referrals; and NHS costs associated with maintenance of the ePROM system.

Where possible, data on NHS resource utilisation will be collected from the electronic patient records. Other data will be collected via CRFs, either completed in study follow-up visits or on event-triggered forms (e.g. generated in response to an ePROM alert). Resource use will be valued using appropriate unit costs such as the British National Formulary and the most recent version of Unit Costs of Health and Social Care and NHS Reference Costs.[29]

For peer review only

For peer review only - http://bmjopen.bmj.com/site/about/guidelines.xhtml

Monitoring

Management and oversight

The Trial Management Group will monitor all aspects of the conduct and progress of the trial, ensure that the protocol is adhered to and take appropriate action to safeguard participants and the quality of the trial itself.

A single Trial Steering Committee (TSC) will be convened and will meet at least yearly and as required depending on the needs of the TSC/trial office. The TSC will provide overall oversight of the trial, including the practical aspects of the study, as well as ensuring that the study is run in a way which is both safe for the patients and provides appropriate feasibility data to the sponsor and investigators. The TSC will also undertake the final assessment of feasibility. Given this is a pilot study, a data monitoring committee is not required.

Adverse events

The collection and reporting of Adverse Events (AEs) will be in accordance with the UK Policy Framework for Health and Social Care Research and the requirements of the Health Research Authority (HRA). The Investigator will assess the seriousness and causality (relatedness) of all AEs experienced by the trial participants and this assessment will be documented in the source data, with reference to the protocol.

Audit

Trials staff will be in regular contact with the site research team to check on progress and address any queries that they may have. Trials staff will check incoming ICFs and CRFs for compliance with the protocol, data consistency, missing data and timing. Sites will be sent data clarification forms requesting missing data or clarification of inconsistencies or discrepancies. Sites will be requested to send in copies of signed ICFs and other documentation for in-house review for all participants providing explicit consent.

Qualitative Sub-study

Objective

To explore patient and study personnel/clinician thoughts/experiences regarding the RePROM trial processes.

Methods

Participants and study personnel/clinicians involved in the pilot trial will be invited to take part in the qualitative sub-study. Up to 40 participants (20 patients and 20 study personnel/clinicians) will be recruited, purposively selected to capture those participants who experienced a range of outcomes and experiences during the trial where possible. However, recruitment will continue until data saturation is reached.

Semi-structured interviews will be conducted by a member of the Centre for Patient Reported Outcome Research (CPROR) research team at the University of Birmingham according to a pre-defined topic guide, but there will be sufficient scope

For peer review only - http://bmjopen.bmj.com/site/about/guidelines.xhtml

to explore novel themes where appropriate. All interviews will be digitally recorded, professionally transcribed and the transcripts anonymised. Transcript data will be entered into a specialist software package (e.g. Nvivo, QSR International) to aid organisation and analysis of the data. All data will be analysed by the CI using conventional content analysis. Formal triangulation of coding and member checking will be employed to enhance the credibility of the analysis. Only anonymised quotes will be used in any arising publications or reports.

ETHICS, CONFIDENTIALITY AND DISSEMINATION

The study has received a favourable opinion from the West Midlands - Edgbaston Research Ethics Committee (Reference: 18/WM/0013). Personal data recorded on all documents will be regarded as strictly confidential and will be handled and stored in accordance with the Data Protection Act 1998. Data transferred from the host site to the researchers (University of Birmingham) will be securely stored and only used for analysis or study monitoring relevant to the participant taking part in the research. The findings from the trial will be used to inform the design of a future, full-scale, RCT. Results will be submitted for publication in a peer reviewed journal, presented at scientific conferences and disseminated to the public via lay summaries/newsletters in partnership with our patient advisory group. Study manuscripts will be prepared by the Chief Investigator (CI*)* and authorship will be determined by the trial publication policy. Intellectual property rights will be addressed in the Clinical Study Site Agreement between Sponsor and site. The full protocol is available on request to the corresponding author.

TRIAL SPONSOR AND FUNDING

This study is sponsored by the University of Birmingham (reference: RG_16-141) and funded by the UK National Institute for Health Research (reference: PDF-2016-09-009). Protocol version 1.1, 07/02/2018.

PATIENT AND PUBLIC INVOLVEMENT

Development of the research question, outcome measures and study design was informed by a series of meetings held with the RePROM patient advisory group (PAG), which included people with lived experience of CKD. The PAG reviewed all patient-facing study documentation and considered the overall burden of study participation during the design process.

For peer review only - http://bmjopen.bmj.com/site/about/guidelines.xhtml

References

1. Fraser SD, Roderick PJ, Aitken G, et al. Chronic kidney disease, albuminuria and socioeconomic status in the Health Surveys for England 2009 and 2010. *Journal of public health* 2013;36(4):577-86.
2. Kerr M, Bray B, Medcalf J, et al. Estimating the financial cost of chronic kidney disease to the NHS in England. *Nephrology Dialysis Transplantation* 2012;27(suppl_3):iii73-iii80.
3. TKHA G. Kidney Health: Delivering Excellence. Kidney Research UK. 2013; [Available at: http://www.kidneyresearchuk.org/file/media/Kidney-Health-Delivering-Excellence-1709-15-Oct.pdf accessed Nov 2015]
4. Calvert MJ, Freemantle N. Use of health-related quality of life in prescribing research. Part 1: why evaluate health-related quality of life? *Journal of Clinical Pharmacy and Therapeutics* 2003;28:513-21.
5. Velikova G, Brown JM, Smith AB, et al. Computer-based quality of life questionnaires may contribute to doctor-patient interactions in oncology. *Br J Cancer* 2002;86(1):51-9. doi: 10.1038/sj.bjc.6600001 [published Online First: 2002/02/22]
6. Detmar SB, Muller MJ, Schornagel JH, et al. Health-related quality-of-life assessments and patient-physician communication: a randomized controlled trial. *Jama* 2002;288(23):3027-34.
7. McCann L, Maguire R, Miller M, et al. Patients' perceptions and experiences of using a mobile phone-based advanced symptom management system (ASyMS©) to monitor and manage chemotherapy related toxicity. *European journal of cancer care* 2009;18(2):156-64.
8. Velikova G, Booth L, Smith AB, et al. Measuring quality of life in routine oncology practice improves communication and patient well-being: a randomized controlled trial. *Journal of Clinical Oncology* 2004;22(4):714-24.
9. Joyce Davidson B, Goldenberg L, Gleave ME, et al. Provision of Individualized Information to Men and Their Partners to Facilitate Treatment Decision Making in Prostate Cancer. *Oncology Nursing Forum* 2003;30(1):107-14.
10. Dy SM, Roy J, Ott GE, et al. Tell Us™: a web-based tool for improving communication among patients, families, and providers in hospice and palliative care through systematic data specification, collection, and use. *Journal of pain and symptom management* 2011;42(4):526-34.
11. Gibson F, Aldiss S, Taylor RM, et al. Involving health professionals in the development of an advanced symptom management system for young people: The ASyMS©-YG study. *European Journal of Oncology Nursing* 2009;13(3):187-92.
12. Kyte D, Draper H, Calvert M. Patient-reported outcome alerts: ethical and logistical considerations in clinical trials. *Jama* 2013;310(12):1229-30. doi: 10.1001/jama.2013.277222 [published Online First: 2013/09/26]
13. Maguire R, McCann L, Miller M, et al. Nurse's perceptions and experiences of using of a mobile-phone-based Advanced Symptom Management System (ASyMS©) to monitor and manage chemotherapy-related toxicity. *European Journal of Oncology Nursing* 2008;12(4):380-86.
14. Snyder C, Jensen R, Orion Courtin S, et al. Patient viewpoint: a website for patient-reported outcomes assessment. *Quality of Life Research* 2009;18:793-800.
15. Snyder CF, Aaronson N, Chouchair AK, et al. Implementing patient-reported outcomes assessment in clinical practice: a review of the options and considerations. *Quality of Life Research* 2011;21(8):1305-14.

For peer review only - http://bmjopen.bmj.com/site/about/guidelines.xhtml

16. Hjollund NHI, Larsen LP, Biering K, et al. Use of patient-reported outcome (pro) measures at group and patient levels: Experiences from the generic integrated pro system, westchronic. *Interactive journal of medical research* 2014;3(1)
17. Acceptability of an online system for reporting and managing symptomatic adverse events (eRAPID): Patients views eRAPID is funded by a National Institute for Health Research (NIHR) Programme Development Grant RP-DG-1209-10031; ISCTRN trial number CCT-NAPN-21338. PSYCHO-ONCOLOGY; 2013. WILEY-BLACKWELL 111 RIVER ST, HOBOKEN 07030-5774, NJ USA.
18. Schick-Makaroff K, Molzahn A. Strategies to use tablet computers for collection of electronic patient-reported outcomes. *Health and quality of life outcomes* 2015;13(1):2.
19. Schick-Makaroff K, Molzahn AE. Evaluation of real-time use of electronic patient-reported outcome data by nurses with patients in home dialysis clinics. *BMC health services research* 2017;17(1):439.
20. Chan A, Tetzlaff J, Altman DG, et al. SPIRIT 2013 Statement: Defining Standard Protocol Items for Clinical Trials. *Annals of Internal Medicine* 2013; http://annals.org/
21. Abernethy AP, Herndon JE, Wheeler JL, et al. Feasibility and acceptability to patients of a longitudinal system for evaluating cancer-related symptoms and quality of life: pilot study of an e/Tablet data-collection system in academic oncology. *Journal of pain and symptom management* 2009;37(6):1027-38.
22. Basch E, Deal A, Kris M, et al. Symptom Monitoring With Patient-Reported Outcomes During Routine Cancer Treatment: A Randomized Controlled Trial. *Journal of Clinical Oncology* 2015;10.1200/JCO.2015.63.0830
23. Hilarius DL, Kloeg PH, Gundy CM, et al. Use of health-related quality-of-life assessments in daily clinical oncology nursing practice. *Cancer* 2008;113(3):628-37.
24. Tangri N, Stevens LA, Griffith J, et al. A predictive model for progression of chronic kidney disease to kidney failure. *Jama* 2011;305(15):1553-59.
25. Kellum JA, Lameire N, Aspelin P, et al. Kidney disease: improving global outcomes (KDIGO) acute kidney injury work group. KDIGO clinical practice guideline for acute kidney injury. *Kidney international supplements* 2012;2(1):1-138.
26. Devlin NJ, Krabbe PF. The development of new research methods for the valuation of EQ-5D-5L. *The European Journal of Health Economics* 2013;14(1):1-3.
27. Lancaster GA, Dodd S, Williamson PR. Design and analysis of pilot studies: recommendations for good practice. *Journal of evaluation in clinical practice* 2004;10(2):307-12.
28. Browne RH. On the use of a pilot sample for sample size determination. *Statistics in medicine* 1995;14(17):1933-40.
29. Curtis L. Unit costs of health and social care 2015. Canterbury, Kent, UK: Personal Social Services Research Unit, University of Kent; 2015, 2016.

For peer review only

For peer review only - http://bmjopen.bmj.com/site/about/guidelines.xhtml

FOOTNOTES

Author Contributions

DK is the chief investigator and takes final responsibility for study design, conduct and decision to submit for publication. DK prepared the first draft of the protocol with approval from all authors. PC, HE, MD, GH and SS provided clinical expertise. EB, NI and MV provided trials expertise. JB (statistician) designed and wrote the statistical analysis section. LJ (health economist) advised on the health economic data collection and analysis. All investigators provided advice and critical input on the study design. All authors critically revised the manuscript for its important intellectual content. All authors read and approved the final manuscript.

Funding
This paper presents independent research funded by the National Institute for Health Research (NIHR) Post-Doctoral Fellowship Scheme, grant number PDF-2016-09-009.

Disclaimer
The views expressed are those of the author(s) and not necessarily those of the NHS, the NIHR or the Department of Health. The study sponsor and funders have no role in study design, including collection, management, analysis and interpretation of data; writing of the report and the decision to submit the report for publication.

Competing interests EB and MV report grants from NIHR. MC receives personal fees from Astellas Pharma and Ferring.

Patient consent Not applicable.

Ethics approval This protocol (V.1.1) was approved by West Midlands - Edgbaston Research Ethics Committee (Ref: 18/WM/0013) on 23rd February 2018.

Acknowledgements
The authors acknowledge Anita Walker for her administrative support and the RePROM patient advisory group for their input into the design of the study.

For peer review only

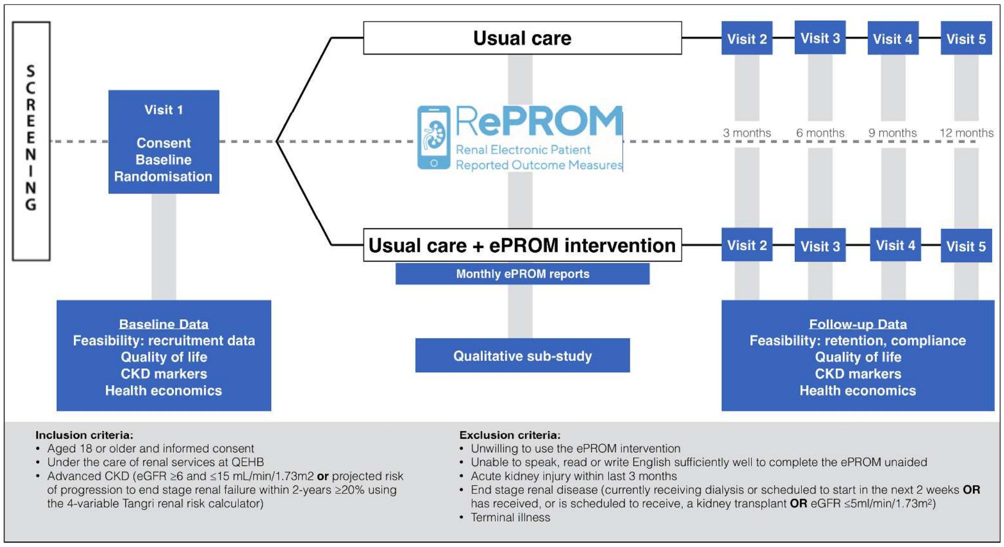

Figure 1. RePROM trial schema.

361x270mm (300 x 300 DPI)

Reporting checklist for protocol of a clinical trial.

Based on the SPIRIT guidelines.

Instructions to authors

Complete this checklist by entering the page numbers from your manuscript where readers will find each of the items listed below.

Your article may not currently address all the items on the checklist. Please modify your text to include the missing information. If you are certain that an item does not apply, please write "n/a" and provide a short explanation.

Upload your completed checklist as an extra file when you submit to a journal.

In your methods section, say that you used the SPIRIT reporting guidelines, and cite them as:

Chan A-W, Tetzlaff JM, Altman DG, Laupacis A, Gøtzsche PC, Krleža-Jerić K, Hróbjartsson A, Mann H, Dickersin K, Berlin J, Doré C, Parulekar W, Summerskill W, Groves T, Schulz K, Sox H, Rockhold FW, Rennie D, Moher D. SPIRIT 2013 Statement: Defining standard protocol items for clinical trials. Ann Intern Med. 2013;158(3):200-207

		Reporting Item	Page Number
Title	#1	Descriptive title identifying the study design, population, interventions, and, if applicable, trial acronym	1
Trial registration	#2a	Trial identifier and registry name. If not yet registered, name of intended registry	2
Trial registration: data set	#2b	All items from the World Health Organization Trial Registration Data Set	See note 1
Protocol version	#3	Date and version identifier	14
Funding	#4	Sources and types of financial, material, and other support	14
Roles and responsibilities: contributorship	#5a	Names, affiliations, and roles of protocol contributors	1
Roles and responsibilities:	#5b	Name and contact information for the trial sponsor	14

For peer review only

sponsor contact information			
Roles and responsibilities: sponsor and funder	#5c	Role of study sponsor and funders, if any, in study design; collection, management, analysis, and interpretation of data; writing of the report; and the decision to submit the report for publication, including whether they will have ultimate authority over any of these activities	17
Roles and responsibilities: committees	#5d	Composition, roles, and responsibilities of the coordinating centre, steering committee, endpoint adjudication committee, data management team, and other individuals or groups overseeing the trial, if applicable (see Item 21a for data monitoring committee)	12
Background and rationale	#6a	Description of research question and justification for undertaking the trial, including summary of relevant studies (published and unpublished) examining benefits and harms for each intervention	4
Background and rationale: choice of comparators	#6b	Explanation for choice of comparators	5
Objectives	#7	Specific objectives or hypotheses	9
Trial design	#8	Description of trial design including type of trial (eg, parallel group, crossover, factorial, single group), allocation ratio, and framework (eg, superiority, equivalence, non-inferiority, exploratory)	8
Study setting	#9	Description of study settings (eg, community clinic, academic hospital) and list of countries where data will be collected. Reference to where list of study sites can be obtained	6
Eligibility criteria	#10	Inclusion and exclusion criteria for participants. If applicable, eligibility criteria for study centres and individuals who will perform the interventions (eg, surgeons, psychotherapists)	7
Interventions: description	#11a	Interventions for each group with sufficient detail to allow replication, including how and when they will be administered	8

For peer review only

Interventions: modifications	#11b	Criteria for discontinuing or modifying allocated interventions for a given trial participant (eg, drug dose change in response to harms, participant request, or improving / worsening disease)	8
Interventions: adherance	#11c	Strategies to improve adherence to intervention protocols, and any procedures for monitoring adherence (eg, drug tablet return; laboratory tests)	11
Interventions: concomitant care	#11d	Relevant concomitant care and interventions that are permitted or prohibited during the trial	n/a
Outcomes	#12	Primary, secondary, and other outcomes, including the specific measurement variable (eg, systolic blood pressure), analysis metric (eg, change from baseline, final value, time to event), method of aggregation (eg, median, proportion), and time point for each outcome. Explanation of the clinical relevance of chosen efficacy and harm outcomes is strongly recommended	9
Participant timeline	#13	Time schedule of enrolment, interventions (including any run-ins and washouts), assessments, and visits for participants. A schematic diagram is highly recommended (see Figure)	6
Sample size	#14	Estimated number of participants needed to achieve study objectives and how it was determined, including clinical and statistical assumptions supporting any sample size calculations	10
Recruitment	#15	Strategies for achieving adequate participant enrolment to reach target sample size	10
Allocation: sequence generation	#16a	Method of generating the allocation sequence (eg, computer-generated random numbers), and list of any factors for stratification. To reduce predictability of a random sequence, details of any planned restriction (eg, blocking) should be provided in a separate document that is unavailable to those who enrol participants or assign interventions	8
Allocation concealment	#16b	Mechanism of implementing the allocation sequence (eg, central telephone; sequentially numbered, opaque, sealed	8

For peer review only

mechanism		envelopes), describing any steps to conceal the sequence until interventions are assigned	
Allocation: implementation	#16c	Who will generate the allocation sequence, who will enrol participants, and who will assign participants to interventions	8
Blinding (masking)	#17a	Who will be blinded after assignment to interventions (eg, trial participants, care providers, outcome assessors, data analysts), and how	6
Blinding (masking): emergency unblinding	#17b	If blinded, circumstances under which unblinding is permissible, and procedure for revealing a participant's allocated intervention during the trial	n/a
Data collection plan	#18a	Plans for assessment and collection of outcome, baseline, and other trial data, including any related processes to promote data quality (eg, duplicate measurements, training of assessors) and a description of study instruments (eg, questionnaires, laboratory tests) along with their reliability and validity, if known. Reference to where data collection forms can be found, if not in the protocol	10
Data collection plan: retention	#18b	Plans to promote participant retention and complete follow-up, including list of any outcome data to be collected for participants who discontinue or deviate from intervention protocols	10
Data management	#19	Plans for data entry, coding, security, and storage, including any related processes to promote data quality (eg, double data entry; range checks for data values). Reference to where details of data management procedures can be found, if not in the protocol	10
Statistics: outcomes	#20a	Statistical methods for analysing primary and secondary outcomes. Reference to where other details of the statistical analysis plan can be found, if not in the protocol	10
Statistics: additional analyses	#20b	Methods for any additional analyses (eg, subgroup and adjusted analyses)	11
Statistics: analysis population and missing data	#20c	Definition of analysis population relating to protocol non-adherence (eg, as randomised analysis), and any statistical methods to handle missing data (eg, multiple imputation)	11

For peer review only

Data monitoring: formal committee	#21a	Composition of data monitoring committee (DMC); summary of its role and reporting structure; statement of whether it is independent from the sponsor and competing interests; and reference to where further details about its charter can be found, if not in the protocol. Alternatively, an explanation of why a DMC is not needed	12
Data monitoring: interim analysis	#21b	Description of any interim analyses and stopping guidelines, including who will have access to these interim results and make the final decision to terminate the trial	12
Harms	#22	Plans for collecting, assessing, reporting, and managing solicited and spontaneously reported adverse events and other unintended effects of trial interventions or trial conduct	13
Auditing	#23	Frequency and procedures for auditing trial conduct, if any, and whether the process will be independent from investigators and the sponsor	13
Research ethics approval	#24	Plans for seeking research ethics committee / institutional review board (REC / IRB) approval	14
Protocol amendments	#25	Plans for communicating important protocol modifications (eg, changes to eligibility criteria, outcomes, analyses) to relevant parties (eg, investigators, REC / IRBs, trial participants, trial registries, journals, regulators)	14
Consent or assent	#26a	Who will obtain informed consent or assent from potential trial participants or authorised surrogates, and how (see Item 32)	7
Consent or assent: ancillary studies	#26b	Additional consent provisions for collection and use of participant data and biological specimens in ancillary studies, if applicable	n/a
Confidentiality	#27	How personal information about potential and enrolled participants will be collected, shared, and maintained in order to protect confidentiality before, during, and after the trial	14
Declaration of interests	#28	Financial and other competing interests for principal investigators for the overall trial and each study site	18
Data access	#29	Statement of who will have access to the final trial dataset,	See

For peer review only

		and disclosure of contractual agreements that limit such access for investigators	note 2
Ancillary and post trial care	#30	Provisions, if any, for ancillary and post-trial care, and for compensation to those who suffer harm from trial participation	n/a
Dissemination policy: trial results	#31a	Plans for investigators and sponsor to communicate trial results to participants, healthcare professionals, the public, and other relevant groups (eg, via publication, reporting in results databases, or other data sharing arrangements), including any publication restrictions	14
Dissemination policy: authorship	#31b	Authorship eligibility guidelines and any intended use of professional writers	14
Dissemination policy: reproducible research	#31c	Plans, if any, for granting public access to the full protocol, participant-level dataset, and statistical code	14
Informed consent materials	#32	Model consent form and other related documentation given to participants and authorised surrogates	n/a
Biological specimens	#33	Plans for collection, laboratory evaluation, and storage of biological specimens for genetic or molecular analysis in the current trial and for future use in ancillary studies, if applicable	n/a

Author notes

1. https://www.birmingham.ac.uk/research/activity/mds/trials/bctu/trials/renal/RePROM/index.aspx
2. Journal System

The SPIRIT checklist is distributed under the terms of the Creative Commons Attribution License CC-BY-ND 3.0. This checklist was completed on 15. August 2018 using http://www.goodreports.org/, a tool made by the EQUATOR Network in collaboration with Penelope.ai

For peer review only

BMJ Open

The use of an electronic Patient-Reported Outcome Measure in the Management of Patients with Advanced Chronic Kidney Disease. The RePROM Pilot Trial Protocol.

Journal:	*BMJ Open*
Manuscript ID	bmjopen-2018-026080.R1
Article Type:	Protocol
Date Submitted by the Author:	06-Sep-2018
Complete List of Authors:	Kyte, Derek; University of Birmingham, Centre for Patient-Reported Outcomes Research, Institute of Applied Health Research<br>Bishop, Jon; NIHR Surgical Reconstruction and Microbiology Research Centre, Medical Statistician<br>Brettell, Elizabeth; 1. Birmingham Clinical Trials Unit (BCTU), Institute of Applied Health Research University of Birmingham, Birmingham, UK<br>Calvert, Melanie; The University of Birmingham, Centre for Patient-Reported Outcomes Research, Institute of Applied Health Research<br>Cockwell, Paul; University Hospitals Birmingham NHS Foundation Trust, Renal Medicine<br>Dutton, Mary; University Hospitals Birmingham NHS Foundation Trust, Renal Medicine<br>Eddington, Helen; University Hospitals Birmingham NHS Foundation Trust, Renal Medicine<br>Hadley, Gabby; University Hospitals Birmingham NHS Foundation Trust, Renal Medicine<br>Ives, Natalie; 1. Birmingham Clinical Trials Unit (BCTU), Institute of Applied Health Research University of Birmingham, Birmingham, UK<br>Jackson, Louise; University of Birmingham, Health Economics Unit<br>Stringer, Stephanie; University Hospitals Birmingham NHS Foundation Trust, Renal Medicine<br>Valente, Marie; 1. Birmingham Clinical Trials Unit (BCTU), Institute of Applied Health Research University of Birmingham, Birmingham, UK
**Primary Subject Heading**:	Renal medicine
Secondary Subject Heading:	Patient-centred medicine
Keywords:	End stage renal failure < NEPHROLOGY, Chronic renal failure < NEPHROLOGY, patient-reported outcomes, symptom monitoring

SCHOLARONE™
Manuscripts

TITLE

The use of an electronic Patient-Reported Outcome Measure in the Management of Patients with Advanced Chronic Kidney Disease. The RePROM Pilot Trial Protocol.

AUTHORSHIP

Derek Kyte[1], Jon Bishop[2], Elizabeth Brettell[2], Melanie Calvert[1], Paul Cockwell[3], Mary Dutton[3], Helen Eddington[3], Gabby Hadley[3], Natalie Ives[2], Louise Jackson[4], Stephanie Stringer[3], Marie Valente[2].

AUTHOR AFFILIATIONS

1. Centre for Patient-Reported Outcomes Research, Institute of Applied Health Research, University of Birmingham, Birmingham, UK
2. Birmingham Clinical Trials Unit (BCTU), Institute of Applied Health Research University of Birmingham, Birmingham, UK
3. University Hospitals Birmingham NHS Foundation Trust, Birmingham, UK
4. Health Economics Unit, Institute of Applied Health Research, University of Birmingham, Birmingham, UK

CORRESPONDING AUTHOR

Dr Derek Kyte
Lecturer in Health Research Methods
The Murray Learning Centre
University of Birmingham
Birmingham
B15 2TT

Telephone: 0121 415 8502
Email: d.g.kyte@bham.ac.uk

WORD COUNT

3994

KEYWORDS

Chronic Kidney Diseases, Patient-reported outcomes, symptom monitoring

For peer review only

ABSTRACT

Introduction

Chronic Kidney Disease (CKD) affects up to 16% of adults in the UK. Patient quality of life is particularly reduced in end stage renal disease and is strongly associated with increased hospitalisation and mortality. Thus, accurate and responsive healthcare is a key priority. Electronic Patient-Reported Outcome Measures (ePROMs) are online questionnaires which ask patients to self-rate their health status. Evidence in oncology suggests the use of ePROM data within routine care, alongside clinical information, may enhance symptom management and improve patient outcomes. However, NHS-based ePROM research in CKD is lacking. This pilot trial will assess the feasibility of undertaking a full-scale randomised controlled trial in patients with CKD within the NHS.

Methods and analysis

The RePROM pilot trial is an investigator-led single-centre, open-label, two-arm randomised controlled pilot trial of 66 participants ≥18 years with advanced CKD. Participants will be randomised to receive either usual care, or usual care supplemented with an ePROM intervention. Participants within the intervention arm will be asked to submit monthly self-reports of their health status using the ePROM system. The system will provide tailored information to patients in response to each report and notify the clinical team of patient deterioration. The renal clinical team will monitor for ePROM notifications and will respond with appropriate action, in line with standard clinical practice. Measures of study feasibility, participant quality of life and CKD severity will be completed at 3-monthly intervals. Health economic outcomes will be assessed. Clinicians will record treatment decision-making. Acceptability and feasibility of the protocol will be assessed alongside outcome measure and intervention compliance rates. Qualitative process evaluation will be conducted.

Ethics and dissemination

The findings will inform the design of a full-scale RCT and the results will be submitted for publication in peer-reviewed journals. The study has ethical approval.

Trial registration number

ISRCTN12669006

For peer review only

ARTICLE SUMMARY

Strengths and limitations of this study

- The study uses a randomised controlled trial design, delivered in an NHS setting.
- The research site, University Hospitals Birmingham NHS Foundation Trust, hosts one of the largest specialist renal research sites in Europe.
- The study protocol and intervention was developed by a multidisciplinary team of experts including: patients, health care professionals and academics.
- As a pilot trial, the study is limited to one clinical centre and is not statistically powered to assess clinical outcomes. Instead, the project will evaluate feasibility of conducting a full-scale RCT in the target population.

For peer review only

INTRODUCTION

Chronic Kidney Disease (CKD) affects up to 16% of adults in the UK[1], with an annual estimated cost of £1.45 billion.[2] More than half of these costs were associated with Renal Replacement Therapy (RRT) - haemodialysis, peritoneal dialysis and/or kidney transplantation - for patients with end stage renal disease (ESRD).[2] This is despite the fact that patients requiring RRT comprise less than 1% of the total CKD population.[2] Numerous studies have demonstrated that patients with ESRD experience high symptom burden and a very high prevalence of depression.[3] Moreover, patient quality of life is significantly reduced in ESRD and is strongly associated with increased hospitalisation and mortality.[3] Accurate and responsive healthcare for patients as they progress from advanced CKD to ESRD is therefore a key healthcare priority.

Effective management of advanced CKD relies on the timely detection of clinical deterioration towards ESRD. This can be a major challenge between scheduled clinic visits, when it is often difficult to identify clinical deterioration unless a patient self-refers. Unfortunately, some patients self-refer too late because they have difficulty identifying the point at which they may require assistance. Without prompt recognition of advanced symptoms, such patients are at high risk of severe illness, emergency hospitalisation, and associated worse clinical outcomes.[3]

Patient-Reported Outcome Measures (PROMs) are validated questionnaires which ask patients to self-rate their health status. They can provide important information regarding the patient's perspective on the physical, functional and psychological consequences of treatment and the degree and impact of disease symptoms.[4] Evidence suggests the use of PROM data, alongside regular clinical information, within routine care may:

- Aid patient-provider communication and support shared decision-making.[5]
- Improve patient activation and help patients to feel more involved/empowered in decisions around their care.[6-8]
- Improve the accuracy of symptom assessment and enhance symptom management.[9]
- Enhance patient education and self-management and maximise patient safety.[7 10-15]

With recent advances in technology, there has been considerable interest in the use of electronic PROMs (ePROMs) for the routine monitoring of patients with long-term conditions. ePROMs offer patients an 'electronic' method of data entry – e.g. web-based via PC, smartphone, or using tablet devices – and give clinicians a flexible platform with which they may view PROM data.[14 16 17]

ePROMs offer patients the option of inputting data at a time and place, and via a platform, that is convenient to them. ePROM data can be used to help provide patients with tailored advice on self-management and can provide clinicians with detailed HRQL and symptom data both in-clinic and between scheduled appointments via home/remote data capture.[15]

For peer review only

For patients with advanced CKD, this would allow clinicians to monitor for symptom deterioration, facilitating the early detection of problems requiring attention and promoting timely intervention from the clinical team (e.g. advice aimed at aiding patient self-management or escalation of care). Such intervention may delay disease progression and the need for costly and invasive RRT, and reduce emergency hospitalisations and other adverse outcomes.

A recent randomised controlled trial (RCT) conducted in an oncology setting in the US, demonstrated that ePROM use is associated with improved HRQL, reduced Accident and Emergency visits, reduced hospitalisations, and superior quality-adjusted survival.[15] Both in Canada and Denmark, ePROM collection has been shown to be feasible in a renal population.[16 18 19] However, NHS-based ePROM research in CKD, utilising real-time patient and clinician feedback is lacking. Routine remote use of ePROM data by patients with advanced CKD may aid self-management, whilst also helping to improve the flow of information between patients and their clinicians, potentially improving patient safety, enhancing clinical interactions, optimising patient outcomes and delivering cost savings to the NHS.

A RCT is needed to evaluate ePROM efficacy in advanced CKD to determine if health professionals, providers and policy-makers should implement routine ePROM collection in renal practice. However, before a definitive trial is undertaken, a pilot trial is required to assess the feasibility of undertaking such a study and to help inform the key elements (e.g. appropriate outcome measure, sample size) of the design for the full-scale RCT.

For peer review only

METHODS

Design
The RePROM study will span two phases: stage 1 involves development of the ePROM intervention with patient and clinician input; stage 2 is a pilot/feasibility RCT with a qualitative sub-study.

Setting
Patients under the care of the renal services at Queen Elizabeth Hospital, Birmingham (QEHB) will be recruited for this study.

Stage 1: Intervention development
Design of the ePROM system will be finalised during a series of operational meetings, held in stage 1 of the study, with regular input from: (1) the QEHB renal clinical and research team; (2) the RePROM Patient Advisory Group; (3) the QEHB IT and Informatics group; (4) the University of Birmingham Clinical Trials Unit; and (5) the Patients Outcomes Group at the University of Leeds.

The ePROM system will be made available to study participants via 'myHealth@QEHB', a secure patient portal linked to the patient's electronic healthcare record (EHR), delivered by the host site. The system will be developed to allow patients to self-report their health status using a variety of electronic platforms, e.g.: PC, smartphone or tablet.

The ePROM system will be designed to: (i) provide appropriate self-management advice to participants whose questionnaire scores suggest mild/moderate symptoms; and (ii) notify the clinical team of patient deterioration via an automated email, where the patient's questionnaire responses indicate severe symptoms/cause for concern.

Patients' longitudinal ePROM scores will be made available to clinicians for use during routine outpatient consultations via the EHR. The RePROM Patient Advisory Group felt this approach would help to focus clinical discussion on patient-centred issues and may enhance symptom management, a view supported by related literature.[7 8 16 17 20-22]

Stage 2: pilot/feasibility RCT and qualitative sub-study

Trial design
The RePROM pilot trial is an investigator led single-centre, open-label, two-arm randomised controlled pilot trial of 66 participants aged 18 years or over with advanced CKD. Participants will be randomised to receive either usual care, or usual care supplemented with an ePROM intervention. The trial is registered with ISRCTN (ISRCTN12669006) and the NIHR Portfolio (CPMS ID: 36497). The study flow is outlined in Figure 1.

Figure 1. RePROM trial schema.

For peer review only

Recruitment and eligibility

Recruitment is scheduled to commence in July 2018. Patients will be invited to participate based on the following eligibility criteria:

Inclusion Criteria

- Aged ≥18 years old;
- Ability to provide fully informed written consent for participation in the study;
- Patients under the care of the renal services at QEHB;
- Patients meeting the trial definition of **advanced CKD**:
    - an eGFR ≥6 and ≤15 mL/min/1.73m^2 (inclusive)

    OR
    - a projected risk of progression to end-stage renal failure within 2-years ≥20% using the 4-variable Tangri renal risk calculator.[23]

Exclusion Criteria

- Patients unwilling to use the ePROM intervention;
- Patients who, in the opinion of the consenting professional, cannot speak, read or write English sufficiently well to complete the ePROM unaided;
- An episode of **acute kidney injury** (defined in accordance with national guidelines) within the last 3 months[24];
- Patients meeting the trial definition of **End Stage Renal Disease**:
    - Currently receiving dialysis or scheduled to start in the next 2 weeks

    OR
    - Has received (or has a scheduled date to receive) a kidney transplant

    OR
    - eGFR ≤5ml/min/1.73m^2;
- A terminal illness that, in the opinion of the consultant assessing eligibility, is likely to lead to the death of the patient within 6 months of starting participation in the study.

Patients fulfilling the entry criteria will have their eligibility assessed by qualified personnel at the host research site with access to/full understanding of their medical history. All patients approached will be given a copy of the participant information sheet (PIS). Usually, this will be sent to the patient in the post, along with an invitation letter, normally in advance of their next clinic assessment. The renal research team may also contact the patient by phone at the time of sending out the PIS. Staff will allow time for potential participants to consider the information provided, discuss the trial with their family and friends, and decide whether to take part. Alternatively, if deemed appropriate by the recruiting renal research team member, the PIS and invitation letter may be provided directly in clinic. Provided the patient feels they have had sufficient time to consider their potential involvement, consent may be sought at this same appointment.

For peer review only

Investigators/delegate(s) will ensure that they adequately explain the aim, trial intervention, anticipated benefits and potential hazards of taking part in the trial to the participant. They will also stress that participation is voluntary and that the participant is free to refuse to take part and may withdraw from the trial at any time. The participant will be given the opportunity to ask questions. If the participant expresses an interest in participating in the trial, they will be asked to sign and date the latest version of the Informed Consent Form.

Trial intervention

Participants in the control arm will continue to receive usual care. Participants randomised to the intervention arm will be asked to commence monthly self-reporting of their health status using the ePROM system, after receiving a face-to-face training session.

Participants will receive automated reminders prior to each scheduled self-report and 24 hours after a failure to report if necessary, these will be delivered via a secure myHealth QEHB patient portal email or text message, according to participants' preferences. Participants may also upload additional ad-hoc reports to the system, if they feel this is necessary (e.g.: if they wish to communicate a sudden change in symptoms).

The ePROM system will provide tailored information to patients in response to each report (both scheduled and ad-hoc) and alert the clinical team of patient deterioration according to *a priori* determined alert threshold criteria established in the intervention development stage. After receiving training during the study setup period, the renal clinical team will monitor for ePROM alerts and will respond with appropriate clinical action, in line with standard clinical practice.

Randomisation

Participants will be randomised at the level of the individual in a 1:1 ratio to either usual care (control arm) or usual care supplemented with an electronic Patient-Reported Outcome Measure (ePROM) system (experimental arm). A minimisation algorithm will be used within the online randomisation system to ensure balance in the treatment allocation over the following variables:

- Risk progression (<40%, versus ≥40%, using the 4-variable Tangri renal risk calculator[23]);
- Self-reported computer experience (defined as: regular use of a computer or tablet or smartphone at least weekly versus less than weekly);
- Ethnicity ('white' versus 'non-white')

A 'random element' will be included in the minimisation algorithm, so that each patient has a probability (unspecified here), of being randomised to the opposite treatment that they would have otherwise received.

Outcome measures and study procedures

Primary outcome

For peer review only

The primary aims of the study are to pilot the trial protocol and assess the feasibility of undertaking a full-scale RCT on the use of ePROMs in the management of advanced CKD. This will include assessment of both quantitative and qualitative data. The pilot study will:

- Test and pilot the trial protocol (including recruitment and retention rates, data collection processes, data completeness and adherence to the ePROM intervention);
- Assess the willingness of clinicians to randomise participants into the trial;
- Assess the willingness of people with advanced CKD to be randomised into the trial;
- Assess the acceptability of the ePROM intervention;
- Explore the need for a non-web-based intervention platform for participants who are unable to use the ePROM;
- Inform selection of the most appropriate primary outcome measure for the full-scale RCT;
- Provide data to help estimate the sample size for the full-scale RCT;
- Provide a platform to develop and pilot the processes to capture costs and outcomes to inform the health economic evaluation for the full-scale RCT;
- Determine key participation criteria for centre involvement in the full-scale RCT.

Outcome data

This pilot trial is not powered to detect differences in outcome measures, but it provides the opportunity to ensure that there are no issues with completion of the outcome data and proposed outcome measures for the main RCT. The following outcome data will be collected:

- HRQL data, using the paper version of the EQ-5D-5L. The EQ-5D-5L is a reliable/validated generic measure of health status/utility commonly used internationally in cost-effectiveness research.[25]
- Clinical data, including: Serum Creatinine, Calcium, Phosphate, Bicarbonate, Albumin, eGFR, ACR, blood pressure, and for participants with diabetes: glucose and HbA1c.
- The following event data: progression to end stage renal disease, contact with health care professionals in secondary care (outpatient clinics and Accident and Emergency); inpatient hospitalisation; death.
- Healthcare resource use data will be collected at each study visit.

All study staff/participants will be invited to complete a trial process questionnaire at the end of the study, which will evaluate aspects surrounding: data collection forms/questionnaires; randomisation procedure; acceptability of the intervention; appropriateness of the frequency of ePROM reporting; alert thresholds and management.

For peer review only

Study procedures

During the baseline visit, patients will have demographic details recorded including age, self-assigned ethnicity, educational status, residential postcode (to derive Index of Multiple Deprivation), self-reported computer experience and medical history.

Patient quality of life will be assessed using a paper version of the EQ-5D-5L questionnaire (not a routine test). This will be completed by the participant at baseline, and at 3, 6, 9 and 12 months post randomisation (assessment window +/- 3 weeks). This questionnaire may be posted out to participants prior to their scheduled clinic/research visit, but research staff will be on hand in clinic to assist with completion where required. The EQ-5D-5L instrument is a reliable/validated generic measure of health status commonly used in CKD trials research.[25]

Clinical data will be collected at baseline, and at 3, 6, 9 and 12 months (assessment window +/- 3 weeks), including: Serum Creatinine, Calcium, Phosphate, Bicarbonate, Albumin, eGFR, ACR, blood pressure, and for participants with diabetes: glucose and HbA1c. Since these measures are routinely collected for clinical monitoring, the results closest to the calculated visit due date will be used for trial data, rather than repeating tests which have already been performed. If a result is not available within the visit window, the test should be performed at the trial visit. Healthcare resource use will be collected at 3, 6, 9 and 12 months (assessment window +/- 3 weeks).

All data will be extracted from the source records and entered onto a secure database maintained by the Birmingham Clinical Trials Unit (BCTU) after each scheduled follow-up visit. The database will automatically capture ePROM notification data. Between clinic visits, patients will be managed in accordance with local practice.

Sample size and justification

As this is a pilot trial, no formal sample size calculation has been performed. Following recommendations for pilot studies, 30 patients or more are typically required to obtain estimates of the parameters needed for sample size estimatation.[26 27] To allow for a 10% drop-out and lost to follow-up rate, this pilot trial will aim to recruit at least 33 participants in each group, a total of 66 participants. This will also allow the recruitment and retention rates to be estimated with 95% confidence interval maximum widths of 20% and 25% respectively.

Analysis of Outcome Measures

A separate Statistical Analysis Plan will be produced and will provide a more comprehensive description of the planned statistical analyses. A brief outline of these analyses is given below.

The primary comparison groups will be composed of those randomised to usual care (control group) versus those randomised to usual care supplemented with the ePROM intervention (experimental group). In the first instance, all analyses will be based on the intention to treat principle, i.e. all participants will be analysed in the treatment group to which they were randomised irrespective of compliance with the allocated treatment or other protocol deviation. The data analysis for this pilot trial

For peer review only

will be descriptive and mainly focus on confidence interval estimation, with no hypothesis testing performed.

Data will be explored to assess the key feasibility aspects of undertaking a full-scale RCT on the use of ePROMs in the management of advanced CKD. Dichotomous feasibility measures, such as the recruitment and retention rates, as well as data completeness will be reported as numbers and percentages. Where appropriate, these values will be summarised across treatment groups.

Adherence with the ePROM intervention will be assessed by calculating the number and percentage of participants who complete the ePROM reports as scheduled. To be considered adherent, participants will need to have submitted their report within 72 hours of the scheduled time-point. Incomplete submissions (i.e. with some questions not answered) will be accepted for the purpose of measuring adherence. Ad-hoc ePROM reports (i.e. those completed outside the scheduled reporting periods) will not contribute to the assessment of adherence, although we will assess the number of ePROM reports completed by each participant.

The pilot data will also help inform the selection of the most appropriate primary outcome measure for the main RCT and provide data to facilitate estimation of the sample size required for the main RCT. Outcome data on HRQL and clinical data are collected at 3, 6, 9 and 12 months post-randomisation. Analysis methods will be chosen according to the data type of the outcome under investigation, in brief:

- *Continuous endpoints (e.g. quality of life)*: These data will be summarised using means and standard deviations, with differences in means with 95% confidence intervals reported. Longitudinal plots of the data over time will also be constructed for visual presentation of the data.
- *Categorical (dichotomous) endpoints (e.g. hospitalisation rates)*: The number of participants and percentages experiencing the event will be summarised between groups.
- *Time to Event endpoints (e.g. time to ESRD, mortality):* The numbers of participants and percentages experiencing the event will be summarised over time between groups. Kaplan-Meier survival curves will be constructed for visual presentation of time-to-event data.

Subgroup Analyses
Descriptive reports of subgroup variables will be limited to the same variables used in the minimisation algorithm. The availability and completeness of data for the subgroup variables will be summarised to assess their appropriateness as minimisation variables for the main trial, but no formal analysis will be undertaken.

Missing data and sensitivity analyses
Every attempt will be made to collect full follow-up data on all study participants; it is thus anticipated that missing data will be minimal. The assessment of missing data is an outcome measure of this pilot trial. If a suitable primary outcome is identified during the pilot trial, the level of missing data will form one component of the assessment of feasibility for a future trial. As this is a pilot trial, no formal sensitivity analysis will be conducted.

For peer review only

Planned interim analysis

As this is a pilot trial, there are no plans for undertaking any interim analyses. However, the Trial Steering Committee (TSC) will have access to recruitment, retention and data collection information.

Planned final analyses

The primary analysis for the trial will occur once all participants have completed the 12 month assessment and corresponding outcome data has been entered onto the trial database, validated as being ready for analysis, and the database locked. This analysis will include data items up to and including the 12 month assessment.

Health economics

In this study, we will develop and pilot methods to capture the costs and outcomes to inform an economic evaluation in the main RCT. This will examine healthcare resource use and outcomes across the two arms of the study.

The primary perspective adopted will be NHS/personal social care; which will focus on healthcare resource use and costs including: renal staff activity in response to ePROM notifications; GP and hospital consultations; in-patient hospitalisation; medications; referrals; and NHS costs associated with maintenance of the ePROM system.

Where possible, data on NHS resource utilisation will be collected from the electronic patient records. Other data will be collected via CRFs, either completed in study follow-up visits or on event-triggered forms (e.g. generated in response to an ePROM alert). Resource use will be valued using appropriate unit costs such as the British National Formulary and the most recent version of Unit Costs of Health and Social Care and NHS Reference Costs.[28]

For peer review only

Monitoring

Management and oversight
The Trial Management Group will monitor all aspects of the conduct and progress of the trial, ensure that the protocol is adhered to and take appropriate action to safeguard participants and the quality of the trial itself.

A single Trial Steering Committee (TSC) will be convened and will meet at least yearly and as required depending on the needs of the TSC/trial office. The TSC will provide overall oversight of the trial, including the practical aspects of the study, as well as ensuring that the study is run in a way which is both safe for the patients and provides appropriate feasibility data to the sponsor and investigators. The TSC will also undertake the final assessment of feasibility. Given this is a pilot study, a data monitoring committee is not required.

Adverse events

The collection and reporting of Adverse Events (AEs) will be in accordance with the UK Policy Framework for Health and Social Care Research and the requirements of the Health Research Authority (HRA). The Investigator will assess the seriousness and causality (relatedness) of all AEs experienced by the trial participants and this assessment will be documented in the source data, with reference to the protocol.

Audit

Trials staff will be in regular contact with the site research team to check on progress and address any queries that they may have. Trials staff will check incoming ICFs and CRFs for compliance with the protocol, data consistency, missing data and timing. Sites will be sent data clarification forms requesting missing data or clarification of inconsistencies or discrepancies. Sites will be requested to send in copies of signed ICFs and other documentation for in-house review for all participants providing explicit consent.

Qualitative Sub-study

Objective
To explore patient and study personnel/clinician thoughts/experiences regarding the RePROM trial processes.

Methods
Participants and study personnel/clinicians involved in the pilot trial will be invited to take part in the qualitative sub-study. Up to 40 participants (20 patients and 20 study personnel/clinicians) will be recruited, purposively selected to capture those participants who experienced a range of outcomes and experiences during the trial where possible. However, recruitment will continue until data saturation is reached.

Semi-structured interviews will be conducted by a member of the Centre for Patient Reported Outcome Research (CPROR) research team at the University of Birmingham according to a pre-defined topic guide, but there will be sufficient scope

For peer review only

to explore novel themes where appropriate. All interviews will be digitally recorded, professionally transcribed and the transcripts anonymised. Transcript data will be entered into a specialist software package (e.g. Nvivo, QSR International) to aid organisation and analysis of the data. All data will be analysed by the CI using conventional content analysis. Formal triangulation of coding and member checking will be employed to enhance the credibility of the analysis. Only anonymised quotes will be used in any arising publications or reports.

ETHICS, CONFIDENTIALITY AND DISSEMINATION

The study has received a favourable opinion from the West Midlands - Edgbaston Research Ethics Committee (Reference: 18/WM/0013). Personal data recorded on all documents will be regarded as strictly confidential and will be handled and stored in accordance with the Data Protection Act 1998. Data transferred from the host site to the researchers (University of Birmingham) will be securely stored and only used for analysis or study monitoring relevant to the participant taking part in the research. The findings from the trial will be used to inform the design of a future, full-scale, RCT. Results will be submitted for publication in a peer reviewed journal, presented at scientific conferences and disseminated to the public via lay summaries/newsletters in partnership with our patient advisory group. Study manuscripts will be prepared by the Chief Investigator (CI*)* and authorship will be determined by the trial publication policy. Intellectual property rights will be addressed in the Clinical Study Site Agreement between Sponsor and site. The full protocol is available on request to the corresponding author.

TRIAL SPONSOR AND FUNDING

This study is sponsored by the University of Birmingham (reference: RG_16-141) and funded by the UK National Institute for Health Research (reference: PDF-2016-09-009). Protocol version 1.1, 07/02/2018.

PATIENT AND PUBLIC INVOLVEMENT

Development of the research question, outcome measures and study design was informed by a series of meetings held with the RePROM patient advisory group (PAG), which included people with lived experience of CKD. The PAG reviewed all patient-facing study documentation and considered the overall burden of study participation during the design process.

For peer review only

References

1. Fraser SD, Roderick PJ, Aitken G, et al. Chronic kidney disease, albuminuria and socioeconomic status in the Health Surveys for England 2009 and 2010. *Journal of public health* 2013;36(4):577-86.
2. Kerr M, Bray B, Medcalf J, et al. Estimating the financial cost of chronic kidney disease to the NHS in England. *Nephrology Dialysis Transplantation* 2012;27(suppl_3):iii73-iii80.
3. TKHA G. Kidney Health: Delivering Excellence. Kidney Research UK. 2013; [Available at: http://www.kidneyresearchuk.org/file/media/Kidney-Health-Delivering-Excellence-1709-15-Oct.pdf accessed Nov 2015]
4. Calvert MJ, Freemantle N. Use of health-related quality of life in prescribing research. Part 1: why evaluate health-related quality of life? *Journal of Clinical Pharmacy and Therapeutics* 2003;28:513-21.
5. Velikova G, Brown JM, Smith AB, et al. Computer-based quality of life questionnaires may contribute to doctor-patient interactions in oncology. *Br J Cancer* 2002;86(1):51-9. doi: 10.1038/sj.bjc.6600001 [published Online First: 2002/02/22]
6. Detmar SB, Muller MJ, Schornagel JH, et al. Health-related quality-of-life assessments and patient-physician communication: a randomized controlled trial. *JAMA* 2002;288(23):3027-34.
7. McCann L, Maguire R, Miller M, et al. Patients' perceptions and experiences of using a mobile phone-based advanced symptom management system (ASyMS©) to monitor and manage chemotherapy related toxicity. *European journal of cancer care* 2009;18(2):156-64.
8. Velikova G, Booth L, Smith AB, et al. Measuring quality of life in routine oncology practice improves communication and patient well-being: a randomized controlled trial. *Journal of Clinical Oncology* 2004;22(4):714-24.
9. Joyce Davidson B, Goldenberg L, Gleave ME, et al. Provision of Individualized Information to Men and Their Partners to Facilitate Treatment Decision Making in Prostate Cancer. *Oncology Nursing Forum* 2003;30(1):107-14.
10. Dy SM, Roy J, Ott GE, et al. Tell Us™: a web-based tool for improving communication among patients, families, and providers in hospice and palliative care through systematic data specification, collection, and use. *Journal of pain and symptom management* 2011;42(4):526-34.
11. Gibson F, Aldiss S, Taylor RM, et al. Involving health professionals in the development of an advanced symptom management system for young people: The ASyMS©-YG study. *European Journal of Oncology Nursing* 2009;13(3):187-92.
12. Kyte D, Draper H, Calvert M. Patient-reported outcome alerts: ethical and logistical considerations in clinical trials. *Jama* 2013;310(12):1229-30. doi: 10.1001/jama.2013.277222 [published Online First: 2013/09/26]
13. Maguire R, McCann L, Miller M, et al. Nurse's perceptions and experiences of using of a mobile-phone-based Advanced Symptom Management System (ASyMS©) to monitor and manage chemotherapy-related toxicity. *European Journal of Oncology Nursing* 2008;12(4):380-86.
14. Snyder C, Jensen R, Orion Courtin S, et al. Patient viewpoint: a website for patient-reported outcomes assessment. *Quality of Life Research* 2009;18:793-800.
15. Snyder CF, Aaronson N, Chouchair AK, et al. Implementing patient-reported outcomes assessment in clinical practice: a review of the options and considerations. *Quality of Life Research* 2011;21(8):1305-14.

For peer review only

16. Hjollund NHI, Larsen LP, Biering K, et al. Use of patient-reported outcome (pro) measures at group and patient levels: Experiences from the generic integrated pro system, westchronic. *Interactive journal of medical research* 2014;3(1)
17. Acceptability of an online system for reporting and managing symptomatic adverse events (eRAPID): Patients views eRAPID is funded by a National Institute for Health Research (NIHR) Programme Development Grant RP-DG-1209-10031; ISCTRN trial number CCT-NAPN-21338. PSYCHO-ONCOLOGY; 2013. WILEY-BLACKWELL 111 RIVER ST, HOBOKEN 07030-5774, NJ USA.
18. Schick-Makaroff K, Molzahn A. Strategies to use tablet computers for collection of electronic patient-reported outcomes. *Health and quality of life outcomes* 2015;13(1):2.
19. Schick-Makaroff K, Molzahn AE. Evaluation of real-time use of electronic patient-reported outcome data by nurses with patients in home dialysis clinics. *BMC health services research* 2017;17(1):439.
20. Abernethy AP, Herndon JE, Wheeler JL, et al. Feasibility and acceptability to patients of a longitudinal system for evaluating cancer-related symptoms and quality of life: pilot study of an e/Tablet data-collection system in academic oncology. *Journal of pain and symptom management* 2009;37(6):1027-38.
21. Basch E, Deal A, Kris M, et al. Symptom Monitoring With Patient-Reported Outcomes During Routine Cancer Treatment: A Randomized Controlled Trial. *Journal of Clinical Oncology* 2015;10.1200/JCO.2015.63.0830
22. Hilarius DL, Kloeg PH, Gundy CM, et al. Use of health-related quality-of-life assessments in daily clinical oncology nursing practice. *Cancer* 2008;113(3):628-37.
23. Tangri N, Stevens LA, Griffith J, et al. A predictive model for progression of chronic kidney disease to kidney failure. *Jama* 2011;305(15):1553-59.
24. Kellum JA, Lameire N, Aspelin P, et al. Kidney disease: improving global outcomes (KDIGO) acute kidney injury work group. KDIGO clinical practice guideline for acute kidney injury. *Kidney international supplements* 2012;2(1):1-138.
25. Devlin NJ, Krabbe PF. The development of new research methods for the valuation of EQ-5D-5L. *The European Journal of Health Economics* 2013;14(1):1-3.
26. Lancaster GA, Dodd S, Williamson PR. Design and analysis of pilot studies: recommendations for good practice. *Journal of evaluation in clinical practice* 2004;10(2):307-12.
27. Browne RH. On the use of a pilot sample for sample size determination. *Statistics in medicine* 1995;14(17):1933-40.
28. Curtis L. Unit costs of health and social care 2015. Canterbury, Kent, UK: Personal Social Services Research Unit, University of Kent; 2015, 2016.

For peer review only

FOOTNOTES

Author Contributions

DK is the chief investigator and takes final responsibility for study design, conduct and decision to submit for publication. DK prepared the first draft of the protocol with approval from all authors. PC, HE, MD, GH and SS provided clinical expertise. EB, NI and MV provided trials expertise. MC provided expertise surrounding PROM trial methodology. JB (statistician) designed and wrote the statistical analysis section. LJ (health economist) advised on the health economic data collection and analysis. All investigators provided advice and critical input on the study design. All authors critically revised the manuscript for its important intellectual content. All authors read and approved the final manuscript.

Funding
This paper presents independent research funded by the National Institute for Health Research (NIHR) Post-Doctoral Fellowship Scheme, grant number PDF-2016-09-009.

Disclaimer
The views expressed are those of the author(s) and not necessarily those of the NHS, the NIHR or the Department of Health. The study sponsor and funders have no role in study design, including collection, management, analysis and interpretation of data; writing of the report and the decision to submit the report for publication.

Competing interests EB and MV report grants from NIHR. MC receives personal fees from Astellas Pharma and Ferring.

Patient consent Not applicable.

Ethics approval This protocol (V.1.1) was approved by West Midlands - Edgbaston Research Ethics Committee (Ref: 18/WM/0013) on 23rd February 2018.

Acknowledgements
The authors acknowledge Anita Walker for her administrative support and the RePROM patient advisory group for their input into the design of the study.

For peer review only

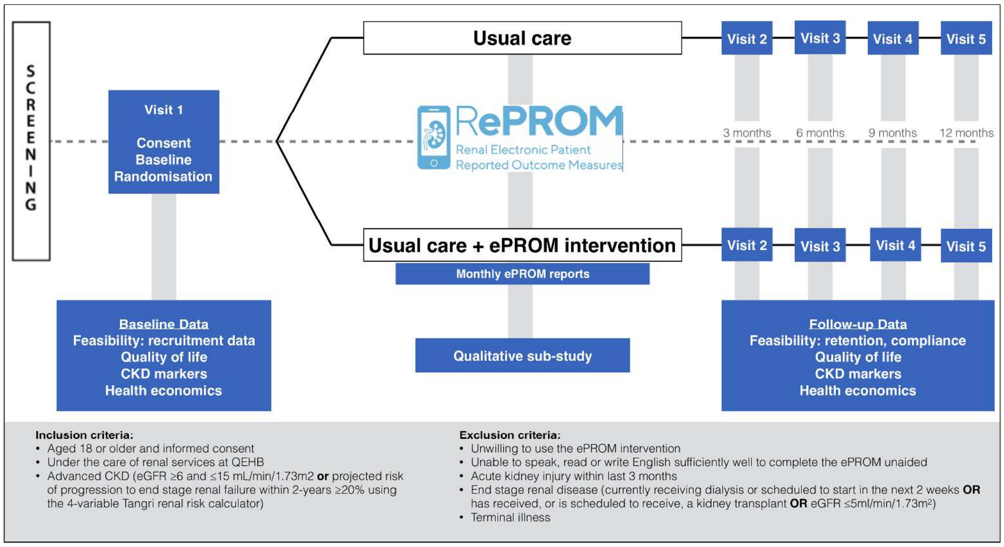

Figure 1. RePROM trial schema.

361x270mm (300 x 300 DPI)

Reporting checklist for protocol of a clinical trial.

Based on the SPIRIT guidelines.

Instructions to authors

Complete this checklist by entering the page numbers from your manuscript where readers will find each of the items listed below.

Your article may not currently address all the items on the checklist. Please modify your text to include the missing information. If you are certain that an item does not apply, please write "n/a" and provide a short explanation.

Upload your completed checklist as an extra file when you submit to a journal.

In your methods section, say that you used the SPIRIT reporting guidelines, and cite them as:

Chan A-W, Tetzlaff JM, Altman DG, Laupacis A, Gøtzsche PC, Krleža-Jerić K, Hróbjartsson A, Mann H, Dickersin K, Berlin J, Doré C, Parulekar W, Summerskill W, Groves T, Schulz K, Sox H, Rockhold FW, Rennie D, Moher D. SPIRIT 2013 Statement: Defining standard protocol items for clinical trials. Ann Intern Med. 2013;158(3):200-207

		Reporting Item	Page Number
Title	#1	Descriptive title identifying the study design, population, interventions, and, if applicable, trial acronym	1
Trial registration	#2a	Trial identifier and registry name. If not yet registered, name of intended registry	2
Trial registration: data set	#2b	All items from the World Health Organization Trial Registration Data Set	See note 1
Protocol version	#3	Date and version identifier	14
Funding	#4	Sources and types of financial, material, and other support	14
Roles and responsibilities: contributorship	#5a	Names, affiliations, and roles of protocol contributors	1
Roles and responsibilities:	#5b	Name and contact information for the trial sponsor	14

For peer review only

sponsor contact information			
Roles and responsibilities: sponsor and funder	#5c	Role of study sponsor and funders, if any, in study design; collection, management, analysis, and interpretation of data; writing of the report; and the decision to submit the report for publication, including whether they will have ultimate authority over any of these activities	17
Roles and responsibilities: committees	#5d	Composition, roles, and responsibilities of the coordinating centre, steering committee, endpoint adjudication committee, data management team, and other individuals or groups overseeing the trial, if applicable (see Item 21a for data monitoring committee)	12
Background and rationale	#6a	Description of research question and justification for undertaking the trial, including summary of relevant studies (published and unpublished) examining benefits and harms for each intervention	4
Background and rationale: choice of comparators	#6b	Explanation for choice of comparators	5
Objectives	#7	Specific objectives or hypotheses	9
Trial design	#8	Description of trial design including type of trial (eg, parallel group, crossover, factorial, single group), allocation ratio, and framework (eg, superiority, equivalence, non-inferiority, exploratory)	8
Study setting	#9	Description of study settings (eg, community clinic, academic hospital) and list of countries where data will be collected. Reference to where list of study sites can be obtained	6
Eligibility criteria	#10	Inclusion and exclusion criteria for participants. If applicable, eligibility criteria for study centres and individuals who will perform the interventions (eg, surgeons, psychotherapists)	7
Interventions: description	#11a	Interventions for each group with sufficient detail to allow replication, including how and when they will be administered	8

For peer review only

Interventions: modifications	#11b	Criteria for discontinuing or modifying allocated interventions for a given trial participant (eg, drug dose change in response to harms, participant request, or improving / worsening disease)	8
Interventions: adherance	#11c	Strategies to improve adherence to intervention protocols, and any procedures for monitoring adherence (eg, drug tablet return; laboratory tests)	11
Interventions: concomitant care	#11d	Relevant concomitant care and interventions that are permitted or prohibited during the trial	n/a
Outcomes	#12	Primary, secondary, and other outcomes, including the specific measurement variable (eg, systolic blood pressure), analysis metric (eg, change from baseline, final value, time to event), method of aggregation (eg, median, proportion), and time point for each outcome. Explanation of the clinical relevance of chosen efficacy and harm outcomes is strongly recommended	9
Participant timeline	#13	Time schedule of enrolment, interventions (including any run-ins and washouts), assessments, and visits for participants. A schematic diagram is highly recommended (see Figure)	6
Sample size	#14	Estimated number of participants needed to achieve study objectives and how it was determined, including clinical and statistical assumptions supporting any sample size calculations	10
Recruitment	#15	Strategies for achieving adequate participant enrolment to reach target sample size	10
Allocation: sequence generation	#16a	Method of generating the allocation sequence (eg, computer-generated random numbers), and list of any factors for stratification. To reduce predictability of a random sequence, details of any planned restriction (eg, blocking) should be provided in a separate document that is unavailable to those who enrol participants or assign interventions	8
Allocation concealment	#16b	Mechanism of implementing the allocation sequence (eg, central telephone; sequentially numbered, opaque, sealed	8

For peer review only

mechanism		envelopes), describing any steps to conceal the sequence until interventions are assigned	
Allocation: implementation	#16c	Who will generate the allocation sequence, who will enrol participants, and who will assign participants to interventions	8
Blinding (masking)	#17a	Who will be blinded after assignment to interventions (eg, trial participants, care providers, outcome assessors, data analysts), and how	6
Blinding (masking): emergency unblinding	#17b	If blinded, circumstances under which unblinding is permissible, and procedure for revealing a participant's allocated intervention during the trial	n/a
Data collection plan	#18a	Plans for assessment and collection of outcome, baseline, and other trial data, including any related processes to promote data quality (eg, duplicate measurements, training of assessors) and a description of study instruments (eg, questionnaires, laboratory tests) along with their reliability and validity, if known. Reference to where data collection forms can be found, if not in the protocol	10
Data collection plan: retention	#18b	Plans to promote participant retention and complete follow-up, including list of any outcome data to be collected for participants who discontinue or deviate from intervention protocols	10
Data management	#19	Plans for data entry, coding, security, and storage, including any related processes to promote data quality (eg, double data entry; range checks for data values). Reference to where details of data management procedures can be found, if not in the protocol	10
Statistics: outcomes	#20a	Statistical methods for analysing primary and secondary outcomes. Reference to where other details of the statistical analysis plan can be found, if not in the protocol	10
Statistics: additional analyses	#20b	Methods for any additional analyses (eg, subgroup and adjusted analyses)	11
Statistics: analysis population and missing data	#20c	Definition of analysis population relating to protocol non-adherence (eg, as randomised analysis), and any statistical methods to handle missing data (eg, multiple imputation)	11

For peer review only

Data monitoring: formal committee	#21a	Composition of data monitoring committee (DMC); summary of its role and reporting structure; statement of whether it is independent from the sponsor and competing interests; and reference to where further details about its charter can be found, if not in the protocol. Alternatively, an explanation of why a DMC is not needed	12
Data monitoring: interim analysis	#21b	Description of any interim analyses and stopping guidelines, including who will have access to these interim results and make the final decision to terminate the trial	12
Harms	#22	Plans for collecting, assessing, reporting, and managing solicited and spontaneously reported adverse events and other unintended effects of trial interventions or trial conduct	13
Auditing	#23	Frequency and procedures for auditing trial conduct, if any, and whether the process will be independent from investigators and the sponsor	13
Research ethics approval	#24	Plans for seeking research ethics committee / institutional review board (REC / IRB) approval	14
Protocol amendments	#25	Plans for communicating important protocol modifications (eg, changes to eligibility criteria, outcomes, analyses) to relevant parties (eg, investigators, REC / IRBs, trial participants, trial registries, journals, regulators)	14
Consent or assent	#26a	Who will obtain informed consent or assent from potential trial participants or authorised surrogates, and how (see Item 32)	7
Consent or assent: ancillary studies	#26b	Additional consent provisions for collection and use of participant data and biological specimens in ancillary studies, if applicable	n/a
Confidentiality	#27	How personal information about potential and enrolled participants will be collected, shared, and maintained in order to protect confidentiality before, during, and after the trial	14
Declaration of interests	#28	Financial and other competing interests for principal investigators for the overall trial and each study site	18
Data access	#29	Statement of who will have access to the final trial dataset,	See

For peer review only

		and disclosure of contractual agreements that limit such access for investigators	note 2
Ancillary and post trial care	#30	Provisions, if any, for ancillary and post-trial care, and for compensation to those who suffer harm from trial participation	n/a
Dissemination policy: trial results	#31a	Plans for investigators and sponsor to communicate trial results to participants, healthcare professionals, the public, and other relevant groups (eg, via publication, reporting in results databases, or other data sharing arrangements), including any publication restrictions	14
Dissemination policy: authorship	#31b	Authorship eligibility guidelines and any intended use of professional writers	14
Dissemination policy: reproducible research	#31c	Plans, if any, for granting public access to the full protocol, participant-level dataset, and statistical code	14
Informed consent materials	#32	Model consent form and other related documentation given to participants and authorised surrogates	n/a
Biological specimens	#33	Plans for collection, laboratory evaluation, and storage of biological specimens for genetic or molecular analysis in the current trial and for future use in ancillary studies, if applicable	n/a

Author notes

1. https://www.birmingham.ac.uk/research/activity/mds/trials/bctu/trials/renal/RePROM/index.aspx
2. Journal System

The SPIRIT checklist is distributed under the terms of the Creative Commons Attribution License CC-BY-ND 3.0. This checklist was completed on 15. August 2018 using http://www.goodreports.org/, a tool made by the EQUATOR Network in collaboration with Penelope.ai

For peer review only
