# Supplementary material for: Use of an electronic patient-reported outcome measure in the management of patients with advanced chronic kidney disease: the RePROM pilot trial protocol
Source: BMJ Open. 2018 Oct 28;8(10):e026080. doi: 10.1136/bmjopen-2018-026080 (PMC6224762; doi:10.1136/bmjopen-2018-026080)
Supplement: Reviewer comments [file bmjopen-2018-026080.reviewer_comments.pdf]

PEER REVIEW HISTORY

BMJ Open publishes all reviews undertaken for accepted manuscripts. Reviewers are asked to complete a checklist review form (**http://bmjopen.bmj.com/site/about/resources/checklist.pdf**) and are provided with free text boxes to elaborate on their assessment. These free text comments are reproduced below.

ARTICLE DETAILS

**TITLE (PROVISIONAL)**	The use of an electronic Patient-Reported Outcome Measure in the Management of Patients with Advanced Chronic Kidney Disease. The RePROM Pilot Trial Protocol.
**AUTHORS**	Kyte, Derek; Bishop, Jon; Brettell, Elizabeth; Calvert, Melanie; Cockwell, Paul; Dutton, Mary; Eddington, Helen; Hadley, Gabby; Ives, Natalie; Jackson, Louise; Stringer, Stephanie; Valente, Marie

This article was not externally reviewed at BMJ Open. Protocols that have been independently assessed prior to submission to BMJ Open are usually fast-tracked to publication on the grounds that further substantial changes will not be possible. This independent assessment will usually be external, independent review for both a substantial grant award from a non-commercial or government funder and ethics approval.
